# Self-assembly of H_2_S-responsive nanoprodrugs based on natural rhein and geraniol for targeted therapy against *Salmonella Typhimurium*

**DOI:** 10.1186/s12951-023-02256-9

**Published:** 2023-12-16

**Authors:** Lu Han, Tao Zang, Lulu Tan, Dunsheng Liang, Tengfei Long, Xuwei Liu, Xiaofan Shen, Hao Ren, ZhiPeng Li, Zhaoxiang Lu, Shengqiu Tang, Xiaoping Liao, Yahong Liu, Chaoqun Zhang, Jian Sun

**Affiliations:** 1 State Key Laboratory for Animal Disease Control and Prevention, South China Agricultural University, Guangzhou, 510642 People’s Republic of China; 2 Guangdong Provincial Key Laboratory of Utilization and Conservation of Food and Medicinal Resources in Northern Region, Henry Fok School of Biology and Agriculture, Shaoguan University, Shaoguan, 512005 People’s Republic of China; 3 Key Laboratory for Biobased Materials and Energy of Ministry of Education, College of Materials and Energy, South China Agricultural University, Guangzhou, 510642 People’s Republic of China; 4 Guangdong Provincial Key Laboratory of Veterinary Pharmaceutics, Development and Safety Evaluation, South China Agricultural University, Guangzhou, 510642 People’s Republic of China; 5 Jiangsu Co-Innovation Center for the Prevention and Control of Important Animal Infectious Diseases and Zoonoses, Yangzhou University, Yangzhou, 225009 People’s Republic of China

**Keywords:** Rhein, Geraniol, PPRG, Self-assembly, H_2_S-responsive nanoprodrugs, *Salmonella* infection

## Abstract

Salmonellosis is a globally extensive food-borne disease, which threatens public health and results in huge economic losses in the world annually. The rising prevalence of antibiotic resistance in *Salmonella* poses a significant global concern, emphasizing an imperative to identify novel therapeutic agents or methodologies to effectively combat this predicament. In this study, self-assembly hydrogen sulfide (H_2_S)-responsive nanoprodrugs were fabricated with poly(α-lipoic acid)-polyethylene glycol grafted rhein and geraniol (PPRG), self-assembled into core–shell nanoparticles via electrostatic, hydrophilic and hydrophobic interactions, with hydrophilic exterior and hydrophobic interior. The rhein and geraniol are released from self-assembly nanoprodrugs PPRG in response to *Salmonella* infection, which is known to produce hydrogen sulfide (H_2_S). PPRG demonstrated stronger antibacterial activity against *Salmonella* compared with rhein or geraniol alone in vitro and in vivo. Additionally, PPRG was also able to suppress the inflammation and modulate gut microbiota homeostasis. In conclusion, the as-prepared self-assembly nanoprodrug sheds new light on the design of natural product active ingredients and provides new ideas for exploring targeted therapies for specific Enteropathogens.

**Graphical Abstract:**

**Figure:**
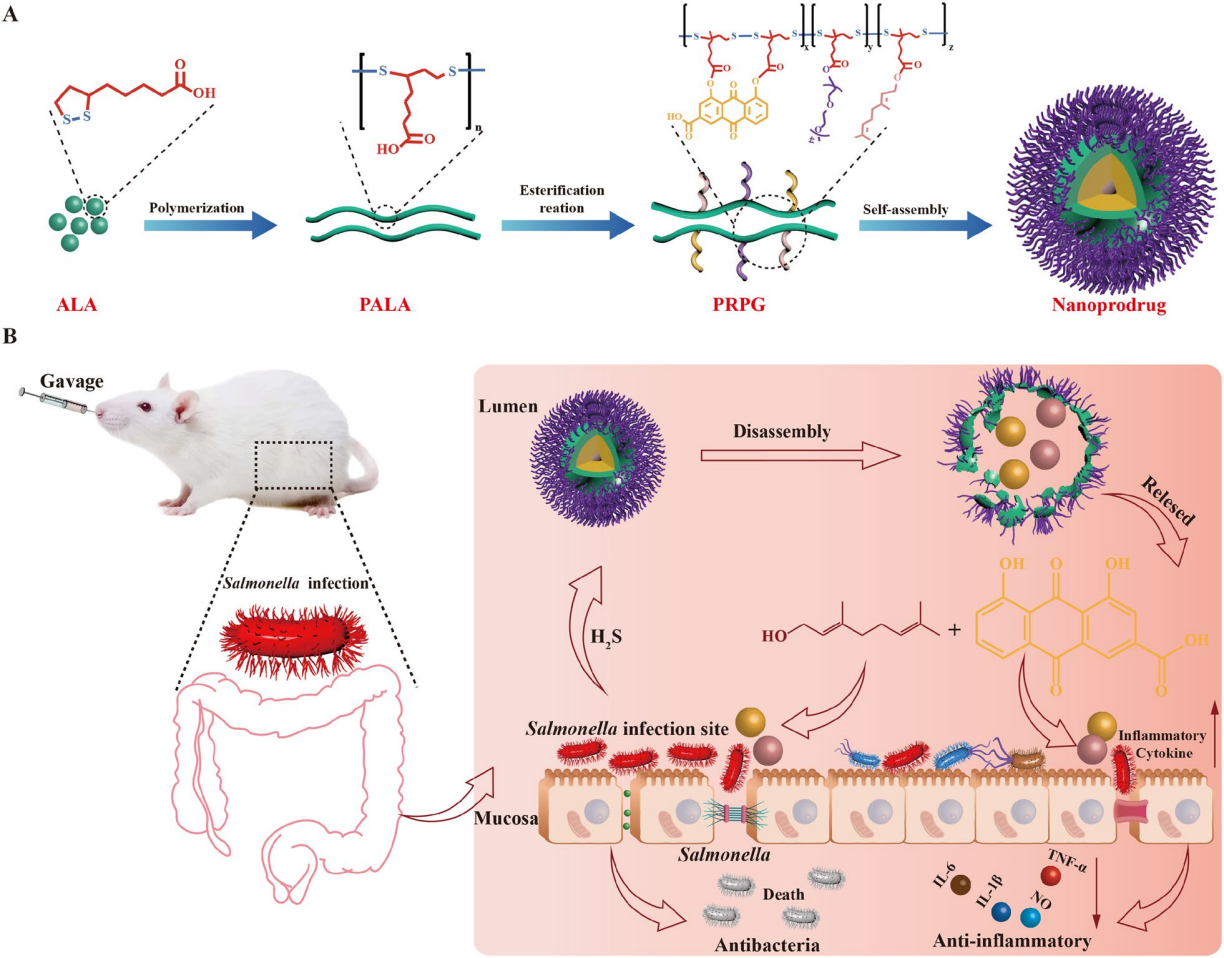
Graphical  illustration for construction of self-assembly nanoprodrugs PPRG and its antibacterial and anti-inflammatory activities on experimental *Salmonella* infection in mice

**Supplementary Information:**

The online version contains supplementary material available at 10.1186/s12951-023-02256-9.

## Introduction

Globally, food-borne diseases are considered as a major public health concern and bring a socioeconomic burden due to their severe mortality [1]. Salmonellosis is one of the most frequently reported food-borne zoonotic diseases, which caused by the Gram-negative Enteropathogens *Salmonella* spp. [2]. Salmonellosis causes 93.8 million cases of gastroenteritis annually, with an estimated 155,000 associated deaths, posing a serious threat to public health [3]. The fluoroquinolones, aminoglycosides, β-lactams, amphenicols, sulfonamides and tetracyclines have been widely used treatment for salmonellosis [2]. Nevertheless, multidrug-resistant (MDR) *Salmonella* disseminates through the food chain, reducing the effectiveness of even newly developed antibiotics against MDR pathogens [4]. Considering the rapid emergence of antimicrobial resistance (AMR) in *Salmonella* spp. [5], innovative antimicrobial agents and novel strategies with unique mechanisms of action against MDR pathogens are urgently needed [6]. Given the laborious, time-consuming, and expensive nature, coupled with high risks, of discovering new antibiotics, exploring alternative approaches derived from Traditional Chinese Medicine (TCM) offers a novel strategy for uncovering the bioactive compounds with the potential to combat bacterial infections [7]. TCM, as an underexplored bioactive compounds resource, has gradually received attentions due to its long history of thousands of years, sound theoretical foundation and rich clinical experience [8].

Rhubarb is one of the most commonly used herbs in TCM and it has been used over thousands of years [9]. The main functional ingredient in rhubarb is anthraquinone including rhein, emodin, aloe-emodin, and chrysophanol. These anthraquinones encompass excellent bioactivity including antitumor, anti-inflammatory, and antimicrobial properties [10]. Rhein, a prominent anthraquinone derived from Chinese herbal medicine rhubarb, has been reported to modulate host inflammation by inhibiting the expression of pro-inflammatory cytokines, including tumor necrosis factor α (TNF-α), interleukin-1β (IL-1β) and interleukin-6 (IL-6) [11]. It is worth mentioning that rhein not only down-regulates the expression of pro-inflammatory cytokines but also up-regulates the expression of anti-inflammatory factors such as interleukin-10 (IL-10) [12]. Additionally, rhein has exhibited antibacterial activity against Gram-positive bacteria [13], and its similar structures have showed antibacterial activity among natural bioactive ingredients against some Gram-negative bacteria [14]. However, the clinical application of rhein was still limited by its side effects including gastrointestinal toxicity and nephrotoxicity [15]. Hence, to enhance the anti-inflammatory activity of rhein, Cai *et al.* synthesized a panel of rhein-non-steroidal anti-inflammatory derivatives through esterification reaction. These derivatives exhibited significantly enhanced anti-inflammatory activity compared to rhein [16]. Yin and his colleagues incorporated rhein into the silk fibroin hydrogels, which exhibited both antibacterial and anti-inflammatory effects [13]. Zheng and his coworkers prepared a series of rhein-based hydrogels through noncovalent self-assembly, indicating excellent stability and sustained release [17]. However, clinical researchers consistently discover that the incorporation of rhein may lead to poor biocompatibility and biodegradability, and the potential of side effects [17]. Additionally, the toxicity of rhein can be determined according to the traditional drug compatibility theory of TCM. Monoterpenes are the major chemical components of essential oils, which were widely presented in TCM for their antimicrobial, antifungal, and antiviral properties. The monoterpene alcohol geraniol, which has been extensively used in treatments based on traditional medicine, is a robust bioactive component [18]. Jang and coworkers reported that geraniol was able to resensitize the efficacy of β-lactams, tetracyclines, and quinolones antibiotics by inhibiting the muti-drug efflux [19]. However, the demerits short in vivo half-life of geraniol remains a challenge for its clinical application [20]. Therefore, Bi *et al.* synthesized geraniol derivatives by the chemical modification of chitosan oligosaccharide onto the amino position, presented a significant inhibition effect of these derivatives on *Escherichia coli* and *Staphylococcus aureus* [21]. Yang and his colleague synthesized geraniol derivatives through modifying the structure of methyl salicylate, and these derivatives exhibited excellent insecticidal activity [22]. Although these approaches can enhance the geraniol effect to some extent, they still have some limitations as internal antibacterial agents.

Nanotechnology is an emerging scientific field widely used in medicine for drug delivery. These carriers include polymeric nanoparticles, liposomes, nanogels, nanoparticles, micelles, dendrimers, etc. [23]. These nanomaterials were designed as precision delivery systems capable of releasing encapsulated bioactive agents in response to specific physiological and pathological changes in pH, temperature, light, ultrasound, reactive oxygen species (ROS), hydrogen sulfide (H_2_S), and enzyme. Yu and colleague prepared a co-delivering and multi-stimuli-responsive nanocarrier using mesoporous silica nanoparticles, which efficiently released the drugs synergistically killing the bacterial cells after stimulated by pathogens [24]. Haas *et al*. synthesized an enzyme-responsive amphiphilic block copolymer system using hyaluronic acid and polycaprolactone. This system can be degraded by hyaluronidase produced by Clostridium and *Streptococcus* spp., resulting in the release of drugs to achieve targeted bacterial eradication [25]. Wang and coworkers developed a facile strategy to fabricate pH and reduction responsive core-crosslinked nanoprodrug micelles [26]. These nanomaterials have inspired us to design a nanoprodrug that can respond to infection-specific cues in vivo.

Stimuli-responsive drug delivery systems have shown potential in bacterial infection therapies. In our previous study, an antibiotic delivery system was synthesized using poly(alpha-lipoic acid) (PALA) and polyethylene glycol (PEG) as efficient H_2_S-responsive carriers encapsulated ciprofloxacin [27]. However, ciprofloxacin still contributes to bacterial AMR by exerting selection pressure. Therefore, looking for natural products or plant derived natural products is an important strategy to reduce selection pressure for AMR. In this study, we found that rhein and geraniol exhibit synergistic antibacterial and anti-inflammatory effects against *Salmonella*. However, the limited oral absorption rate of rhein and geraniol restrict their effectiveness when used the combination in vivo. Thus, we linked rhein and geraniol with PEGylated PALA through ester bonds to obtain H_2_S-stimuli-responsive nanoprodrugs, which can simultaneously release rhein and geraniol at H_2_S-rich sites (Graphical abstract). The efficacy of this nanoprodrug containing rhein and geraniol was validated for the precise eliminate of the target bacteria via stimuli-responsive in vitro and in vivo. These nanoprodrugs were also evaluated in the study to shed the light on the potency of the rhein and geraniol combination for further application in human and animals.

## Results

### Design, synthesis, and characterization of poly(α-lipoic acid)-polyethylene glycol grafted rhein and geraniol (PPRG) nanoprodrug

The effect of rhein with geraniol in combination on the growth of Gram-negative model bacteria, *S. Typhimurium* ATCC 14028, was investigated in the microplates. These results indicated that a typical synergistic effect between the candidate rhein and geraniol were observed with FICI at 0.5 ± 0.072 (Additional file 1: Figure S1A). To reinforce the notion of synergism between the candidate rhein and geraniol, a direct synergistic bactericidal assay was conducted. In the monotherapy assay, the application of rhein and geraniol alone exhibited limited bactericidal activity over time. In contrast, the combination of rhein and geraniol rapidly eradicated the *S. Typhimurium* strains, leading to a reduction in bacterial loads by 10^3^–10^8^-fold within 4 h after treatments (Additional file 1: Figure S1B).

**Fig. 1:**
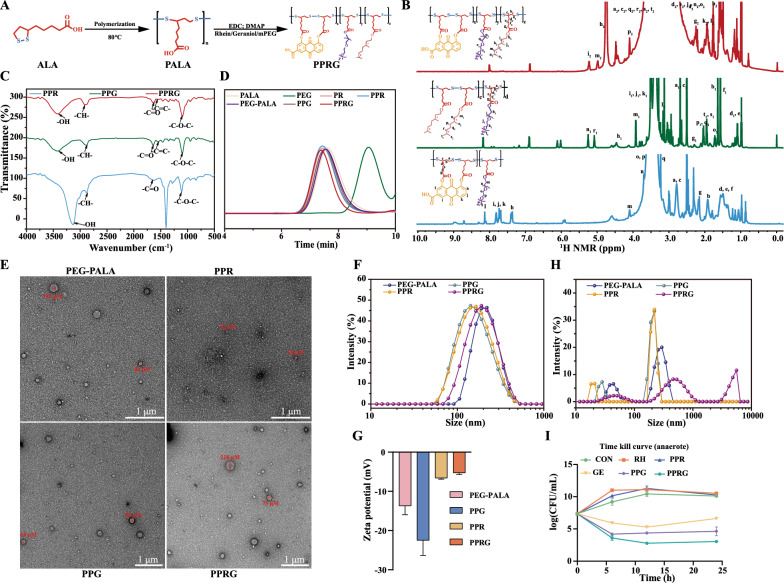
Synthesis and characteristics of PPR, PEGylated PALA, and PPRG. **A** Structural formula of PPRG. **B** ^1^H NMR. **C** FTIR. **D** GPC. **E** TEM. **F** particle size. **G** zeta potential. **H** nanoparticles deal with 1 mM·L^−1^ Na_2_S determined by Zetatrac particle size analyzer of PEGylated PALA, PPR, PPG, and PPRG. (I) Time-killing curve of *S. Typhimurium* ATCC 14028 under anaerobic condition for 24 h. (*CON* control, *SAL* *Salmonella*, *RH* rhein, *PPR* PEGylated PALA grafted rhein, *GE* geraniol, *PPG* PEGylated PALA grafted geraniol, and *PPRG* poly(α-lipoic acid)-polyethylene glycol grafted rhein and geraniol, respectively)

The poly(α-lipoic acid)-polyethylene glycol grafted rhein and geraniol (PPRG) nanoprodrug, was prepared by covalently conjugating poly (α-lipoic acid) and PEG to the hydroxyl (-OH) groups of rhein and geraniol via ester bond as a linker. The synthesis processes of PPRG are shown in Fig. 1A and Additional file 1: Figure S2. The ^1^H NMR spectrum of PPRG reveals the presence of eight distinct types of hydrogen atoms, with additional proton peaks from geraniol denoted as h_2_, i_2_, j_2_, k_2_, l_2_, m_2_, n_2_ and o_2_ (δ4.75, δ5.24, δ1.56, δ1.94, δ1.78, δ4.99, δ1.49 and δ1.48 ppm, respectively), grafted onto PEGylated PALA grafted rhein (PPR) skeleton (Fig. 1B). In the FTIR spectrum, when compared to those of PPR, PEGylated PALA grafted geraniol (PPG), PPRG significant shifts were observed. Specifically, two important bonds corresponding to the ether bond (-C–O–C-) and the -OH groups were exhibited at 1060 cm^−1^ and 3200 cm^−1^. The intensity of -OH group in PPRG decreased in comparison to that of PPR and PPG, while the intensity of -COOR increased in comparison to that in PPR and PPG. The peaks at 1096 cm^−1^ and 3133 cm^−1^ further validated the structure of PPRG (Fig. 1C). Additionally, the molecular weight of PPRG was determined to be 88674 g·mol^−1^, which is greater than that of PPR, indicating that 59.3% geraniol grafted in the PALA skeleton (Fig. 1D). These results confirm the successful synthesis of PPRG. The ^1^H NMR spectra of PR, PPR and PPG demonstrate successful preparation of these compounds.

X-ray photoelectron spectra (XPS) analysis provided the chemical oxidation state of elements present in the samples. The S_2p_ spectrum of the PPR demonstrated the presence of C-S and S–S bonds (161.38 and 163.28 eV), respectively. While the S_2p_ spectrum of the PPRG showed the C-S and S–S bonds (161.48 and 163.28 eV), which represented the PALA present in PPR and PPRG. The C-S bonds of PPR and PPRG are shifted, which also indicates the significantly changes in bond structure (Additional file 1: Figure S3). X-ray diffraction (XRD) patterns further examine on the existence of cross-linking reaction on PALA (Additional file 1: Figure S4), and the crystalline state of α-lipoic acid at 22.8º is almost transformed into the amorphous state [28]. The PPR contained PEG and rhein displayed additional peaks at 19.2º and 23.4º, 27.4º, respectively [29]. Geraniol containing in PPRG does not change the peak of PPR, which is similar to other study [30]. These results further confirm the successful synthesis of PPRG.

HPLC–UV analysis at 258 nm and 210 nm indicated that the content of rhein and geraniol in PPR and PPG were 15.81 ± 5.5% and 48 ± 0.12%, respectively. In the PPRG, the content of rhein and geraniol was determined to be 10.56% ± 1.39% and 13.85% ± 0.87%, respectively. The rhein and geraniol from PPR, PPG and PPRG exhibited hardly released in both simulated gastric fluid (SGF) and simulated intestinal fluid (SIF). To trigger the release of these as-prepared nanoprodrugs, they were allocated into solutions containing dithiothreitol (DTT) served as an in vitro H_2_S donor, following methods described in previous studies [31, 32]. Rhein and geraniol were almost completely released within 2 h in PPR and PPG nanoprodrugs at 1 mM·L^-1^ DTT solution. The PPRG nanoprodrug released 68.6% rhein and almost all of geraniol within 4 h (Additional file 1: Figure S5). Then, the assembled morphology of these nanoprodrugs formed by PEGylated PALA, PPR, PPG and PPRG indicated particle sizes about 50–200 nm, and PPR was the smallest at 50 nm examined by TEM (Fig. 1E). The average sizes ranged from 142 to 220 nm and the zeta potentials ranged from − 22.5 ± 2.67 to − 5.30 ± 0.30 mV (Fig. 1F–G). These nanoprodrugs exhibited excellent storage stability no observable precipitation or delamination was observed after centrifuged at 3500 rpm for 10 min. And the drug content remained almost unchanged in the room temperature after a 14-day storage period (Additional file 1: Figure S5C).

The as-prepared nanoprodrugs were observed to be decomposed in response to sodium sulfide (Na_2_S) as a H_2_S donor (Fig. 1H). To further examine whether culture of the nanoparticles with *S. Typhimurium* would yield bactericidal effect through the release of rhein and geraniol, time-killing curves were generated under anaerobic cultivation to mimic the in vivo intestinal environment. As expected, the bacterial numbers significantly decreased by 4.31log_10_(CFU·mL^−1^) after treatment with PPRG for 24 h. In contrast, PPG treatment resulted in a reduction of 2.72 and 1.99 log_10_(CFU·mL^−1^) compared to the control and geraniol treatments, respectively. This demonstrates a significant effect of the combination of rhein and geraniol (Fig. 1I).

### Permeability assay, in vitro cell viability, and anti-inflammatory activity evaluation

**Fig. 2:**
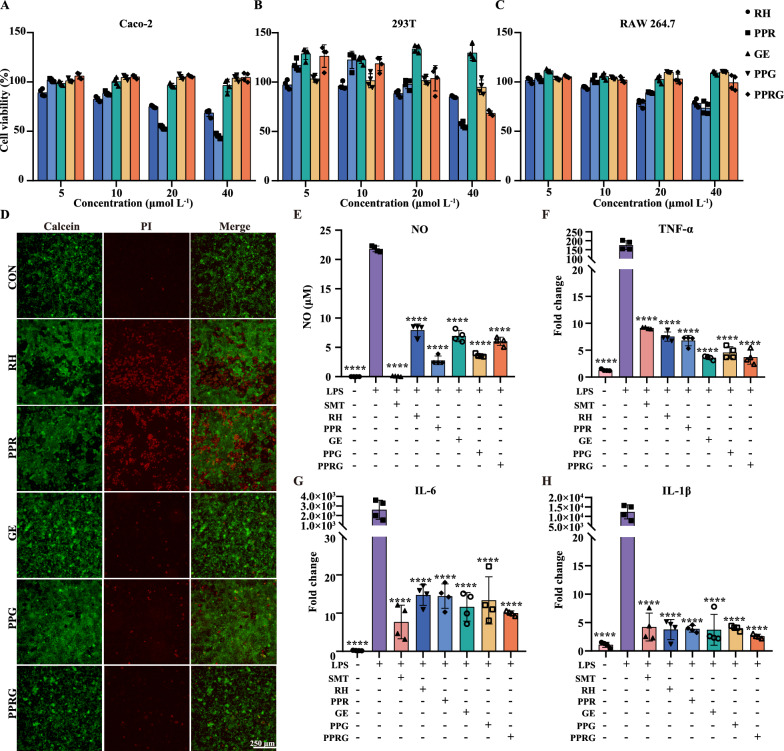
The cell viability and LPS-stimulated RAW 264.7 cellls. Cell viability for **A** Caco-2. **B** 293 T.and **C** RAW 264.7. **D** Live/dead cell staining of Caco-2 cells using 40 μM·L^−1^ test samples. LPS-stimulated RAW 264.7 cells incubated with test samples and measurements of **E** NO. **F **TNF-α. **G** IL-6. and **H** IL-1β

To investigate the biosafety of these nanoprodrugs, cell viability was determined using the cell counting kit-8 (CCK-8). The viability of Caco-2, 293 T, RAW 264.7, HepG2, HGF-1 were examined by adding rhein, PPR, geraniol, PPG, PPRG to the culture medium at concentrations ranging from 5 to 40 μmol·L^−1^, respectively. Cell viability ratio was normalized with the viability of DMSO-treated cells. At a concentration of 40 μmol·L^−1^, the cell viability of Caco-2 exposed to rhein, geraniol, PPR, PPG and PPRG were 63.91, 96.39, 45.34, 103.92, and 104.75%, respectively. Rhein and PPR exhibited low toxicity, especially at concentrations over 20 μmol L^−1^ (Fig. 2A). For 293 T cells, the viability exposed to rhein, geraniol, PPR, PPG, PPRG at 40 μmol L^−1^ were 85.10, 56.72, 129.50, 94.84, and 68.36% (Fig. 2B). RAW 264.7 cell viability remained above 70% across the tested concentration range (Fig. 2C). Hep G2 and HGF-1 cells exhibited significantly reduced viability upon exposure to high doses of rhein and PPR, but no changes in cells viability were observed with other treatments (Additional file 1: Figure S6A–B). Additionally, the live/dead cell staining for Caco-2 and 293 T at 40 μmol·L^−1^ indicated minimal toxicity for rhein and PPR (Fig. 2D, Additional file 1: Figure S6C). These above results showed that PPRG effectively reduces the cytotoxicity of rhein.

Considering the effect of PPRG on the transport of intestinal epithelial cells, a Caco-2 cell Transwell model [33] was established to explore the intestinal permeability features of rhein, PPR, geraniol, PPG and PPRG. The Trans-Epithelial Electrical Resistance (TEER) was measured at 226.7 ± 3.1 Ω /cm^2^ (Additional file 1: Figure S7A). The result showed that the PPR, PPRG, PPG and PPRG significantly decreased the permeability compared to rhein and geraniol (Additional file 1: Figure S7B-7C). The as-prepared compounds at 10 μmol·mL^−1^, which shows not adversely affected of cells, were selected to evaluate the anti-inflammatory effect. The inflammatory process is activated by lipopolysaccharide (LPS)-stimulated, leading to the secretion of nitric oxide (NO) and cytokines such as IL-1β, IL-6, and TNF-α. To evaluate the anti-inflammatory efficacy of the PPRG nanoprodrug, the NO, TNF-α, IL-6, and IL-1β were determined using an LPS-induced RAW 264.7 model to evaluate the expression of pro-inflammatory cytokine expression levels. Curcumin (CUR), well-documented for its ability to reduce NO production and its anti-inflammatory and antioxidant properties, served as a positive control in the study [34]. All the test compounds exhibited significant inhibition of NO production when compared to cells treated with LPS alone (*P* < 0.0001). Compared with rhein treatment, PPR, PPG and PPRG exhibited a significant reduction in NO production (*P* < 0.01), whereas geraniol demonstrated no substantial difference (Fig. 2E). Compared with the LPS-stimulated alone, all the treatments were significantly down-regulated the production of pro-inflammatory cytokines TNF-α, IL-6, and IL-1β. However, compared with rhein or geraniol, CUR, PPR, PPG and PPRG did not exhibit significant differences (Fig. 2F–H). To sum up, these results indicated that PPRG maintained a high-level anti-inflammatory activity while reducing the toxicity of rhein.

### In vivo therapeutic efficacy of the PPRG nanoprodrug against *Salmonella* infection in mice

**Fig. 3:**
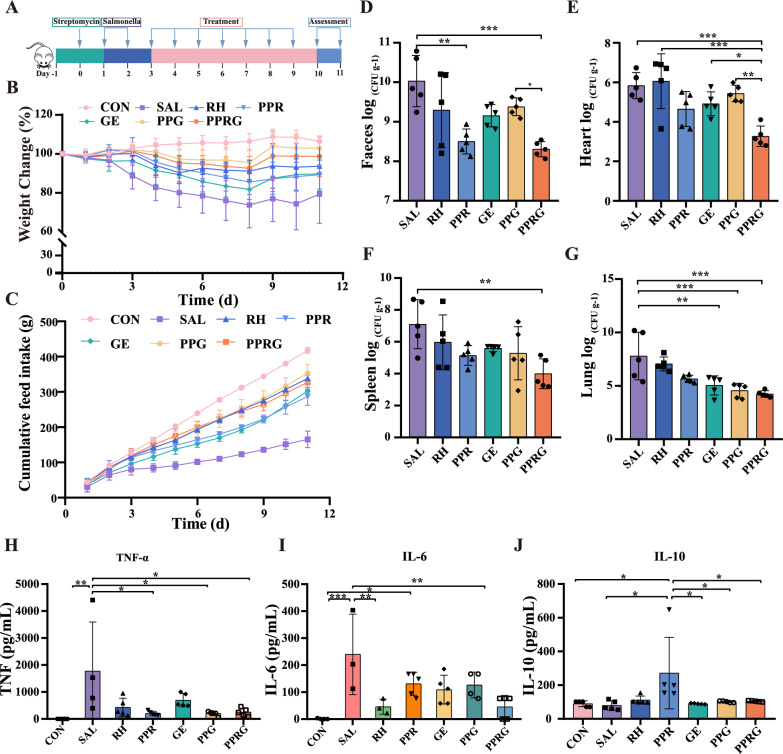
Therapeutic efficacy in an acute *Salmonella* infection in vivo model. **A** The study protocol included streptomycin pretreatment followed by *Salmonella* infection, treatment with rhein (25 mg·kg^−1^), PPR (250 mg·kg^−1^), geraniol (12.5 mg·kg^−1^), PPG (26 mg·kg^−1^) and PPRG (21 mg·kg^−1^), daily. **B** Variations of mouse body weights over time and normalized to the percentage of day zero body weight. **C** Cumulative food intake of per group from day 1 to 11. Quantification of bacterial burdens in **D** faeces. **E** heart. **F** spleen. and **G** lungs of infected mice with different treatments. Serum inflammatory factor levels of **H** TNF-α. **I** IL-6. **J** IL-10 in vivo for *Salmonella*-infected mice

**Fig. 4:**
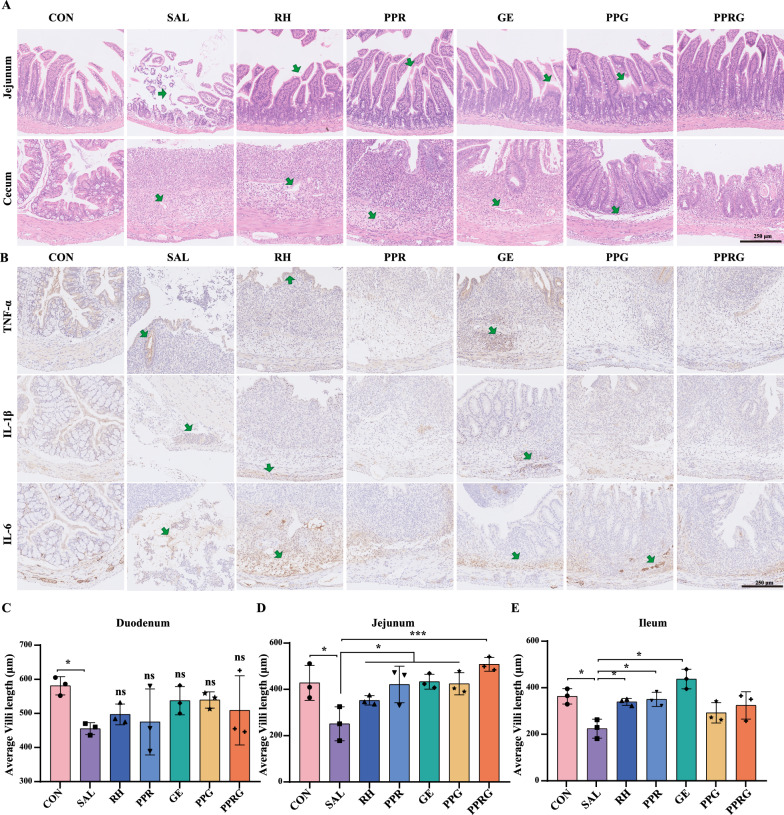
Intestinal morphology of mice in treatment. **A** The H&E staining of the jejunum and cecum in mice. **B** Histologic and immunohistochemical features of the cecum. **C**–**E** Villus length in the small intestine (duodenum, jejunum, and ileum)

To evaluate the potential effect of PPRG in vivo, an acute *Salmonella* infection mouse model was constructed (Fig. 3A). PPRG significantly alleviated infection-induced reduction in body weight compared to *Salmonella* group (*P* < 0.0001), whereas PPG alone did not significantly improve in body weights (*P* = 0.29) (Fig. 3B–C). The bacterial load of *Salmonella* in the feces, hearts, spleens, and lungs were significantly reduced by 1.72, 2.56, 3.11 and 3.55log_10_ CFU· g^−1^ after receiving PPRG treatment. These results demonstrate the substantial efficacy of the PPRG in eradicating *Salmonella* from infected hosts (Fig. 3D–G). *Salmonella* colonization in the feces, hearts, spleens, and lungs were reduced by 1.52, 1.18, 1.96, 2.13log_10_CFU·g^−1^ and 0.65, 0.40, 1.82, 3.24 log_10_ CFU·g^−1^, respectively, after receiving PPR and PPG treatment. These results indicated that the PPRG nanoprodrug exhibited a synergistic effect in vivo by releasing rhein and geraniol after *Salmonella* infection. As to the effects on modulating intestinal inflammatory response, PPRG was observed to significantly down-regulate the level of pro-inflammatory cytokines TNF-α and IL-6. Besides, PPRG notably elevated the levels of the anti-inflammatory factor IL-10, which plays a crucial role in modulating the host inflammatory (Fig. 3H–J). To further investigate the influence of *in situ* effectiveness of the PPRG nanoprodrug at infection sites, immunohistochemistry analysis to assess the expression of TNF-α, IL-1β, and IL-6 in the ceca were conducted. Geraniol treatment up-regulated the expression of inflammatory factors TNF-α and IL-1β, while rhein treatment up-regulated expression of IL-6. PPRG treatment down-regulated the pro-inflammatory cytokines TNF-α, IL-1β, and IL-6 in the ceca compared with *Salmonella* group (Fig. 4B).

To further assessment the histopathologic changes after *Salmonella*, H&E (hematoxylin and eosin) staining was performed. Compared with the control group, PPRG treatment showed reduced tissue destruction caused by *Salmonella* in the jejunum and cecum. Specifically, *Salmonella* infection mice exhibited a significantly thinner serosal layers with evidence of breakage, loss, and irregular arrangement of intestinal villi. In contrast, rhein, PPR, geraniol, PPG, and PPRG treatment showed varied lengths of intestinal villi. PPRG treatment resulted in a significant reduction in the severity of infection in the jejunal tissues compared to *Salmonella* infection (Fig. 4A and D). Moreover, the cecal walls appeared thinner and the muscularis propria was disrupted in the *Salmonella*, rhein, PPR or geraniol group. Goblet cells in the submucosa of PPG- and PPRG-treated mice were fewer compared to the control groups, and the boundary between the mucous and submucosa was indistinct (Fig. 4A, Additional file 1: Figure S8). The jejunal, cecal and colonic tissues in the PPRG group closely resembled those of the control group (Fig. 4D). Interestingly, the PPR group displayed significantly increased villi lengths in the ileum (Fig. 4E). In contrast, *Salmonella* infection led to a significantly decrease in small bowel villi length in the duodenum. And the rhein, PPR, geraniol, PPG or PPRG treatment had a restorative effect on the length of small intestinal villi, although these changes were no statistically significant differences (Fig. 4C and Additional file 1: Figure S8).

### Gut microbiota homeostasis of the PPRG nanoprodrug treatment

**Fig. 5:**
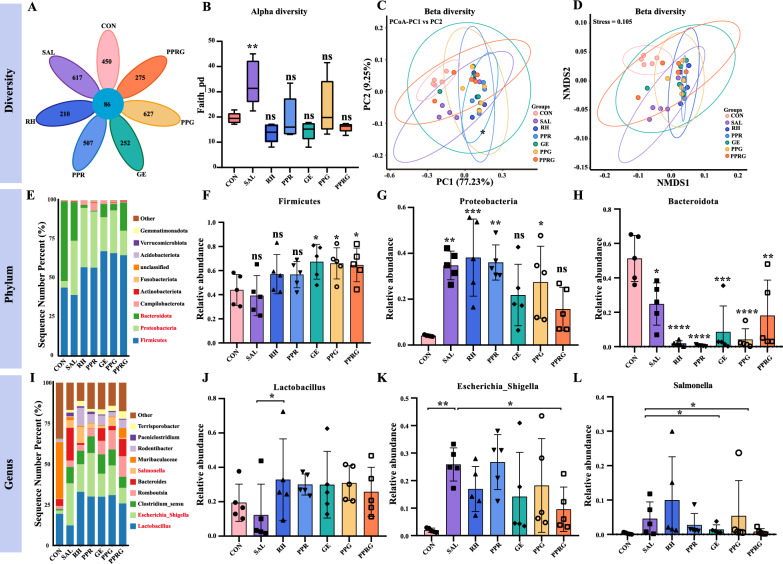
Gut microbiota analysis of infected mice after treatments. **A** Venn diagram indicating different OTU number for the four treatment groups. **B** Average bacterial taxonomic profiling of the gut microbiota in seven groups. **C** Nonmetric multidimensional scaling score plot based on Bray–Curtis distances. **D** UniFrac-based PCoA score plot based on weights. **E** Average bacterial taxonomic profiling of the gut microbiota in seven groups at phylum level. **F**–**H** Relative abundance of bacterial phylum obtained from LefSe results. **I** Average bacterial taxonomic profiling of the gut microbiota in seven groups at the genus level. **J**–**L** Relative abundance of the bacterial genera obtained from the LefSe results

To further assess whether PPRG nanoprodrugs were able to maintain the gut microbiome homeostasis, ceca samples from 7 groups were collected. A total of 5322 operational taxonomic units (OTUs) were identified, with 210 to 627 OTUs being specific to group received different treatments, and 86 OTUs being shared among all 7 groups. Compared to the control group, an increment in OTUs was observed in mice received treatment, whereas the RH or PPRG treatment decreased the microbiota abundances (Fig. 5A). The taxonomic analysis identified a total of 4618 taxa at the phylum level, 4596 at the class level, 4453 at the order level, 3626 at the family level, 1826 at the genus level, and 507 at the species level. Compared with the uninfected mice, the *Salmonella* infection reduced the gut microbial α-diversity (Faith index) (*P* < 0.01) at species abundance level (Fig. 5B). UniFrac distance-based principal Coordinate Analysis (PCoA) and non-metric multidimensional scaling (NMDS) plots indicated that microbiota composition was centralized in rhein and geraniol treatment groups, which led to a reduction to microbiota diversity compared to the PPRG treatment group (Fig. 5C, D). Linear discriminant analysis Effect Size (LEfSe) is commonly utilized for comparing the predominant gut microbiota among different groups. The abundance analysis revealed that the Firmicutes, Proteobacteria, and Bacteroidetes were the most dominant phyla in the gut microbiota. These phyla were likely vulnerable to *Salmonella* infection, leading to alterations in the phylum-level structure of the microbial community. Firmicutes and Proteobacteria significantly increased at expense of Bacteroidetes in comparison to the control group (Fig. 5E). Specifically, the abundance of Firmicutes significantly increased in response to PPRG compared to control group (*P* = 0.08). Compared to the control group, the abundance of Proteobacteria was significantly increased in the *Salmonella*, rhein, PPR, geraniol and PPG groups (*P* = 0.0002, *P* < 0.0001, *P* = 0.0001, *P* = 0.0001, *P* = 0.02, *P* = 0.0029, respectively), while no significant difference was observed in the PPRG groups (*P* = 0.12, Fig. 5F). In addition, *Salmonella* infection led to significant increase in the abundance of Proteobacteria and Bacteroidetes, but PPRG treatment was effective in reversing these alteration microbiota (Fig. 5G–H). Moreover, *Salmonella* infection treatment with drugs significantly increased the abundance of various genera of Enterobacteriaceae. To further define whether PPRG was able to maintain gut microbiota at genus level, the gut microbiota at genus level were analyzed. As shown in Fig. 5I, *Escherichia*_*Shigella*, *Clostridium*, *Bacteroides*, *Romboutsia* and *Salmonella* were significantly increased, while *Lactobacillus* and *Muribaculaceae* were significantly decreased. *Salmonella* infection receiving rhein treatment can significantly increase in the relative abundance of *Lactobacillus* spp. (*P* = 0.047, Fig. 5J)*,* while PPRG treatment sharply decreased the relative abundance of *Escherichia*_*Shigella* caused by *Salmonella* infection (*P* = 0.024). *Salmonella* infection receiving PPRG and GE treatments significantly decreased the relative abundance of *Salmonella* (*P* = 0.049, *P* = 0.0353), which indicated the therapeutic effect of PPRG in vivo.

### The Morris water maze test, pharmacokinetic and pharmacodynamic evaluation of PPRG

To comprehensively assess the effects of the nanoprodrug on the subjects, we conducted pre-treatment, spatial acquisition, and probe trial sessions in the Morris water maze (see Additional file 1: Figure S9A). Our findings indicated that there were no significant changes in weight during the early days of oral administration (Additional file 1: Figure S9B). The spatial learning and memory abilities of the rats subjected to drug pre-treatment were evaluated using the Morris water maze experiment. These results revealed that the spatial learning abilities of mice with rhein, geraniol, or PPRG were no significant effect (Additional file 1: Figure S9C). Compared with the control group, the swimming distance, swimming time and the number of times of swimming across in the target quadrant of mice were no significant changes (Additional file 1: Figure S9D-9H). These results indicated that the rhein, geraniol or PPRG treatment had no significant effect on the cognition and behavior of mice.

Plasma pharmacokinetics was evaluated after oral (70 mg/kg) administration in SD rats [35]. Plasma samples of PPRG, rhein, and geraniol were analyzed at preset time points. After PPRG administration, plasma concentration of rhein and geraniol increased gradually within 5 h (C_max-rhein_, 0.85 ± 0.03 µg/mL; C_max-geraniol_, 155.20 ± 43.9 µg/mL), then declined to 0.22 ± 0.037 and 59.81 ± 28.4 0.46 μg/mL within 8 h, and remained mostly constant up to 24 h. The calculated pharmacokinetic/pharmacodynamic (PK/PD) parameters are shown in Additional file 1: Table S3 and Figure S10. Compared to PPRG, plasma concentration of rhein administration alone increased gradually within 45 min (C_max_, 3.28 ± 0.17), then declined to 0.64 ± 0.086 μg/mL within 3 h. Plasma concentration of geraniol administration alone increased gradually 104.32 ± 11.35 μg/mL within 45 min, then declined to nearly 0 within 3 h. These data have been compared with the reported data for free rhein and geraniol [20, 35]. The comparison showed that it takes long time to reach C_max_ at oral administration of PPRG. Similarly, half-life (t_1/2_) of PPRG was much longer, showing over fourfold and threefold increase compared to free rhein and geraniol. The decrease in plasma concentration of rhein from PPRG showed that the gastrointestinal tract absorbed rhein at a slower rate compare to free rhein. Increased plasma concentration of geraniol from PPRG is a clear indication of the lower clearance from circulation compared to free geraniol. Furthermore, we observed higher t_1/2_, t_max_, and MRT values compared PPRG to free rhein or geraniol. Also, the significant decrease in area under the concentration–time curve (AUC) values for rhein from PPRG were observed compared to free rhein (25.61 ± 18.88 vs 5.73 ± 0.53  μg/h/mL). While the geraniol from PPRG was displayed significantly increased in AUC values compared to free geraniol. These results indicated that PPRG nanoprodrug can effectively maintain T_max_ of absorption and distribution for rhein and geraniol, contributing to enhance their synergy.

## Discussion

For targeted eradication of enteropathogen deeply reside in intestine like *Salmonella*, various applications have been developed. For instance, Mudakavi and coworkers developed an arginine-based nanocarriers to target the intracellular *Salmonella*, which induce the drug release from encapsulation from scavenging the coated arginine [36]. Moreover, Mu et al. established glycovesicles that sense the *Salmonella* produced H_2_S for in situ antibiotic release, which are able to eliminate *Salmonella* infection with high specificity [37]. Inspired by this study, our previous work reported a precision delivery system facilitated by PEGylated poly (lipoic acid) nanocarrier, to enrich the drug concentration at infection sites by specifically responding to the *Salmonella*-associated microenvironments [27]. These studies fully demonstrated the attractive advantages of precision medicine, by which maximally sustained the gut microbiome homeostasis during antibiotic treatments. However, there are still some concerns need to be addressed. First, high dose of antibiotics at infection sites may easily trigger the evolution of antibiotic resistance. Second, the LPS-containing bacterial debris from rapid cell death might induce severe inflammation to the infected hosts [38]. To cope with these, the PPRG designed in this study to treat the bacterial infection with phytobiotic prodrug which could kill bacteria with low selective pressure while alleviate the over-activated inflammatory response. Comparing with the aforementioned investigations, rhein and geraniol are able to synergistically mitigate the *Salmonella* infection with less opportunity for leading to resistance, while enjoying the anti-inflammatory activity of geraniol to profoundly protect gut barrier and epithelial integrity. PPRG nanoprodrug can effectively maintain T_max_ of absorption and distribution for rhein and geraniol, contributing to enhance their synergy. This nanoprodrug is further employed to explore its anti-inflammatory and antimicrobial effects, aiming to assess its potential in alleviating adverse impact on gut microbiota composition caused by *Salmonella* infection.

By establishing a well-established acute *Salmonella* infection model, our findings indicate that the PPRG nanoprodrug may impede the rapid absorption within the small intestine and release the rhein and geraniol at *Salmonella* infection sites. The released rhein and geraniol were then taken up by nearby *Salmonella* pathogens, resulting in a significantly decrease of bacterial burden. The application of PPRG significantly reduced the bacterial load and suppressed the inflammation in the tested animals, which collectively alleviated the pathogenesis caused by infection. In addition, after the PPRG cleaved from the disulfide bond, the released rhein exerted anti-inflammatory effects in vivo [39]. The stomach, duodenum and cecum are the most common sites for the formation of ulcers caused by food-borne *Salmonella* infections [40]. The duodenal mucosal lining has been frequently reported to less respond to the anti-inflammatory medications, which may potentially explain the lack of efficacy of rhein on such tissue [41]. Sal*monella* infection dramatically altered the gut microbial communities as previously reported [42], yet utilization of rhein and geraniol were able to prevent the gut microbiome dysbiosis caused by *Salmonella* infection [39]. The nanoprodrug protected the gut microbiome homeostasis largely depending on its nature in stimuli-responsive releasing [37, 43]. By performing LEfSe analysis, we screened the top 6 species from phylum to genus level that exhibited the most significant differences in gut microbiome after different treatment, and they were mostly found in Firmicutes, Proteobacteria, and Bacteroidetes phyla. In addition, the relative abundance of *Lactobacillus*, *Escherichia_Shigella*, and *Salmonella* genus was compared, indicating PPRG treatment significantly decreased the relative abundance of *Escherichia_Shigella* and *Salmonella*. In contrast, rhein treatment significantly increased the relative abundance of *Lactobacillus*. These results are also consistent with previous reports [44]. *Enterobacteriaceae* in the gut is associated with Crohn’s disease and can induce inflammation and intestinal barrier disruptions [45]. Geraniol decreased Proteobacteria abundance led to a more balanced gut microbiota even in the face of a *Salmonella* infection challenge [39]. PPRG nanoprodrug may not exhibit comparable efficacy to antibiotics like ciprofloxacin etc. in eradicating *Salmonella*, but the PPRG nanoparticles significantly increased the antibacterial activity compared with rhein and geraniol. Moreover, the releasing rhein and geraniol are uptaken by *Salmonella* pathogens nearby and dramatically decrease bacterial burden.

In light of arising demand for precision medicine, PPRG in current study was designed to target the *Salmonella* infection by sensing specific metabolic cues of *Salmonella* [37]. This feature contributed to controlled release of antibacterial agents to sites where the pathogens enriched, and maximally reduced the unnecessary exposure of such agents to commensal microbiota. In this case, the resistances are less expected to be developed. Another advantage of the as-prepared PPRG was its ability to suppress the bacterial infection and associated inflammation at the same time. These benefits in vivo application to efficiently and systematically alleviate the pathogenesis caused by pathogen and promote the rapid recovery from the infection. However, there are still several limitations for the current study. First, the rhein and geraniol herein exhibited the different PK parameters in vivo, indicating that these two drugs might not be exert beneficial effects in a synchronized manner. Thus, the optimization of this nanoprodrug should be prioritized in the coming future. Second, comparing with conventional antibiotics, the rhein is still lack of efficacy to eradicate *Salmonella*, especially in the severe infection model. Hence, our next goal is to improve its therapeutic potential by rational design of deviates with higher efficiency. Third, although the biosafety of nanoprodrug has been proved in vitro, the in vivo biocompatibility still remains unclear. It still calls for further efforts to elaborate whether there are potential side effects for this nanoprodrug.

## Conclusion

In summary, an H_2_S-responsive nanoprodrug of PPRG was developed to selectively target *Salmonella*, the salmonellosis causative pathogen. PPRG nanoprodrug can be readily cleaved by *Salmonella* produced H_2_S, leading to the synergistic release of rhein and geraniol. It exhibited facile cleavage upon exposure to H_2_S produced by *Salmonella*, resulting in the concomitant liberation of rhein and geraniol and the emergence of synergistic effects in vitro and in vivo. Furthermore, it was discovered that the utilization of PPRG nanoprodrug inhibited the expression of pro-inflammatory factors TNF-α and IL-6, alleviation of intestinal inflammation, and modulation of the inflammatory reaction. More importantly, the maintenance of intestinal homeostasis was significantly facilitated by the PPRG nanoprodrug. This research sheds new light on the antibacterial effect of active ingredients of traditional Chinese medicine expected to as an alternative to antibiotics, and suggested a proof-of-principle approach for specifically combatting enteropathogens in a responsive manner and revealed novel strategies for precision medicine.

## Experimental section

### Drug combination sensitivity test in vitro

Overnight *S. Typhimurium* ATCC 14028 were diluted 0.1% in Mueller Hinton (MH) broth, and incubated for 4 h at 37 °C and shaking at 180 rpm. Both drugs were twofold diluted in medium with an initial concentration of 0.64 mg/mL, and mixed with an equal volume of bacterial suspensions (10^6^ colony-forming units (CFU)/mL) in microtiter plate. Then, the optical density at 600 nm (OD_600_) values were used to calculate the fractional inhibitory concentration index (FICI) value. Antibacterial kinetic assay was used to investigate the bactericidal effects of the combination of rhein and geraniol. *S. Typhimurium* ATCC 14028 was diluted tenfold then treated with rhein, geraniol, and rhein combined geraniol at 2.5 mg mL^−1^. The bacterial count (CFU/mL) were checked at 6, 12 and 24 h from100 μL aliquots that were diluted and re-plated on MacConkey agar.

### Synthesis of PEGylated PALA

PALA and PEGylated PALA were synthesized as follows. Alpha -Lipoic acid (ALA, 2.06 g, 10 mmol) was polymerized under nitrogen protection at 80℃ for 2 h. Then, the crude product of PALA was obtained after purification and vacuum drying. The PEGylated PALA was synthesized by conjugation of PEG onto PALA backbone. PEG (2.0 g, 1 mmol) and PALA (824 mg, 1 mmol) were reacted using EDC·HCl (383 mg, 2 mmol) and DMAP (61.08 mg, 0.5 mmol) catalysis for 48 h. Afterwards, products were purified by dialysis (MWCO 3500 Da) in double-distilled water (ddH_2_O) for 72 h, lyophilized to obtain the white powder.

### Synthesis of PPR

PALA (1.6 g, 7.75 mmol) was reacted with rhein (1.1 g, 3.88 mmol) in the presence of EDC**·**HCl (0.74 g, 3.88 mmol) and DMAP (0.12 g, 0.97 mmol) catalysis in a mixed solvent of DMSO and THF under 50 °C for 48 h. Then, products were dialyzed against ddH_2_O, and were suction-filtered and vacuum dried at 50 °C for 24 h to obtain the rhein grafting poly (α-lipoic acid) (PR) yellow–brown solid. PR (0.49 g, 1 mmol) and PEG (2 g, 1 mmol) were catalyzed by EDC·HCl (0.388 g, 2 mmol) and DMAP (0.061 g, 0.5 mmol) at 50℃ in a mixed solvent of DMSO and THF for 48 h. The resulting product was purified by dialysis (MWCO 3500 Da) against ddH_2_O for 72 h, yielding a brown powder after lyophilization for 48 h.

### Synthesis of PPG

PALA, PEG and geraniol were catalyzed by EDC**·**HCl and DMAP (PALA: PEG: geraniol: EDC·HCl: DMAP = 1: 0.2: 0.8: 1: 0.25) in DMF solvent for 48 h, then purification and lyophilization to obtain a white powder.

### Synthesis of PPRG

Geraniol was grafted onto PPR in DMF solvent for 48 h, and the light brown powder was obtained after lyophilization and purification.

### Structural Characterization

The composition of the clusters and the carbon–sulfur interactions of these samples were probed by X-ray photoelectron spectroscopy (XPS). The bonding characterization and morphology were measured by X-ray diffraction (XRD) and transmission electron microscopy (TEM) [25]. The size and zeta potential were examined using Zeta-sizer Nano ZSE instrument.

### Rhein and geraniol loading

The 15 mg of PPR, PPG, PPRG were dissolved in 4 mL DMF by sonication and diluted tenfold, passed through 0.22 μm poresize microporous membrane. The drug loading content (DLC) of rhein and geraniol were determined by HPLC with detection at 258 nm and 210 nm, respectively. The compounds were eluted in a methanol, acetonitrile and 0.1% aqueous phosphoric acid (*v/v/v* = 39:20:41) for rhein and acetonitrile and water (*v/v* = 4:1) for geraniol at 1.0 mL min^−1^ at 30 °C.

### Synthesis of PEGylated PALA, PPR, PPG, PPRG Nanoparticles and the rhein and geraniol release rate in vitro

The PEGylated PALA, PPR, PPG, PPRG (80 mg) were dissolved in 4 mL DMF by ultrasonication, respectively. Then added dropwise to 20 mL phosphate-buffered saline (PBS) with stirring for 2 h and dialyzed against ddH_2_O for 24 h, the nanoparticles were obtained following lyophilization. DTT as the source of H_2_S were used as the release media. SGF, SIF and DTT solutions were prepared as our previous study [27].

### Particle size, zeta potential, and transmission electron microscopy (TEM)

The nanoparticles were dissolved in PBS at concentration of 1 mg mL^−1^ with 0 or 1.0 mmol L^−1^ Na_2_S solution, shaken at 37 °C for 24 h, then size and zeta potential were examined using Zeta-sizer Nano ZSE instrument. Morphologies of PPR, PPG, and PPRG nanoparticles were determined by TEM as previously described [27]. In brief, the PEGylated PALA, PPG, PPR, and PPRG nanoparticles were dropped onto a 200-mesh copper grid and stained with uranyl acetate. Then, TEM images were obtained following deposition and drying.

### In vitro time-kill assay of PPRG nanoparticles

Overnight *S. Typhimurium* ATCC 14028 was diluted 0.1% in Luria–Bertani (LB) broth, and incubated for 4 h at 37 °C, 180 rpm. The bacteria were diluted tenfold then treated with rhein, geraniol, PPR, PPG, and PPRG at equal concentration of 2.5 mg·mL^−1^ using *S. Typhimurium* in anaerobic broth medium and cultured under anaerobic conditions. The initial bacteria 2.26 × 10^7^ CFU/mL were added in 4 mL anaerobic broth. Bacteria with the same volume PBS was used as control. The bacterial count (CFU/mL) were checked at 6, 12 and 24 h from100 μL aliquots that were diluted and re-plated on MacConkey agar.

### In vitro cell viability assay

The CCK-8 assay, live/dead viability assay kit was used to assess the cell viability. The 293 T, Caco-2, RAW 264.7, Hep G2, and HGF-1 cells were seeded in 24-well plate at a determined cell density of about 1 × 10^5^ cells per well in 500 μL of complete DMEM containing 10% FBS and 1% penicillin/streptomycin and incubated at 37 ℃ in 5% CO_2_ for 24 h. Then samples were added into each well reaching different concentrations, while control group contained same volume of serum-free medium. After incubation for 24 h, the medium was removed, washed with sterile PBS, and fresh medium containing 10% CCK-8 was added. The plates were incubated at 37 ℃ for 4 h and 100 μL of cell culture medium was pipetted into a 96-well plate. Absorbance values were measured at 450 nm with a microplate reader to calculate the cell viability. The relative cell viability (%) was calculated by the following formulas:

Cell viability (%) = (A_sample_-A_blank_)/(A_control_- A_blank_) × 100.

A_sample_: Absorbance of wells with cells, CCK-8 solution, and as-prepared drugs solution;

A_blank_: Absorbance of wells with CCK-8 solution;

A_control_: Absorbance of wells with cells and CCK-8 solution.

Cell viability was also assessed using the live and dead cell staining kit for Caco-2 and 293 T cells. These cells were incubated at 37 ℃ for 24 h, washed with sterile PBS, added 250 μL detection buffer containing 0.1% Calcein-AM/PI. Then, the plate was incubated at 37 ℃ for 30 min. Cell images were observed using a fluorescence microscope and the live/dead cells were counted in five randomly selected visual fields.

### Permeability across Caco-2 cells

The as-prepared nanoprodrugs permeability was evaluated using the Caco-2 cell line as an epithelial cell monolayer model. Caco-2 cells were seeded at a density of 1 × 10^4^ cells/cm^2^ in translucent membrane inserts for 21 days. TEER was measured with an epithelial voltammeter Millicell-ERS (Millipore Corporation, MA) to determine the integrity of monolayer. The transport assays were carried out only on monolayers with a TEER above 200 Ω/cm^2^ [46]. Drug solutions were added to apical and basolateral compartments of the monolayer to evaluate apical to basolateral and basolateral to apical transport.

### In vitro evaluation of inflammatory responses and RT-qPCR Analysis

The RAW 264.7 was cultured in complete DMEM containing 10% FBS and 1% penicillin/streptomycin incubation at 37 ℃ in a 5% CO_2_ atmosphere. Cells were seeded in 12-well plates at 1 × 10^6^ cells per well for 12 h. The tested samples were added LPS at 1 μg mL^−1^ for 2 h, then DMEM containing 5 μmol L^−1^ compounds were replaced, incubated for 18 h at 37 ℃. Griess reagent were mixed with 100 μL, incubated at room temperature for 15 min, measured absorbance at 540 nm. A nitric oxide synthase inhibitor S-methylisothiourea (SMT), was used as a positive control.

Determination of the effect of these compounds on the production of inflammatory factors in RAW 264.7 cells by RT-qPCR analysis. Cells were treated with test samples incubated at 37 ℃ for 3 h and then treated with 1 μg mL^−1^ LPS. After incubated for 6 h, total RNA was extracted with an Invitrogen TRIzol (Carlsbad, CA, USA) and reverse transcribed using 1 μg a total RNA to produce complementary DNA (cDNA) for quantitative qRT-PCR using a SensoQuest cDNA Synthesis Kit (Göttingen, Germany). The real-time RT-qPCR reactions were performed using Bio-Rad SYBR Green and cDNA amplification was performed for 30 cycles on the CFX Connect instrument.

### In vivo* Salmonella*-infected mouse model

The 4-week-old female Kunming mice were purchased from Hunan Laike Jingda Experimental Animal, reared in cages at 25 °C and a relative humidity of 40% with water and food ad libitum for two weeks. Following this period, the experimental mice were treated with 20 mg/mouse of streptomycin designated as day (d) 0. After 24 h, mice were infected orally by gavage with 2 × 10^9^ CFU·mL^−1^ of *S. Typhimurium* at 1–2 d. Mice in the treatment groups were treated intragastrically with 100 μL PBS, rhein, PPR, geraniol, PPG, and PPRG on days 3–9. Healthy status of mice was monitored, after an observation period of 2 days, then mice were euthanized. The organs including heart, liver, spleen, and lungs, and intestinal faeces were collected, weighed, homogenized with PBS, and total *S. Typhimurium* colonization were determined using MacConkey agar plates.

### Histological and immunohistochemical analyses

The intestinal segments including duodenum, jejunum, ileum, cecum, and colon were excised, fixed in 4% paraformaldehyde. Then, these intestinal segments were dehydrated using an ethanol series, embedded them in paraffin and 4 μm sections were stained with H&E. Histological alterations were assessed by light microscopy using a Nikon TE2000U microscope (Tokyo, Japan).

Ceca were dissected from mice and fixed in fresh 4% paraformaldehyde solution for 48 h. Paraffin-embedded ceca were sectioned at 4 μm. The extent of inflammatory responses in ceca were assessed by immunohistochemical analysis using commercial Anti-IL-6 polyclonal antibody (Solarbio, China), Anti-TNF-α polyclonal antibody (Thermo Fisher, USA), Anti-IL-10 antibody (Bioss, China). In brief, tissue sections were deparaffinized, rehydrated and heat-treated for antigen retrieval and incubated with antibodies at 4 ℃ overnight, washed with PBS. Secondary antibodies were applied to the tissue for 1 h at room temperature and 0.05% 3, 3-diaminobenzidene followed by hematoxylin was used to stain the slide. The slides were viewed by light microscopy (Tokyo, Japan).

### Gut microbiota profiling

Microbial DNA was extracted from stool samples using a stool DNA extraction kit (Invitrogen, USA). The V3–V4 hypervariable regions of bacterial 16S rDNA were then amplified using commercial primer and the microbial diversity analysis was performed as described [27].

### Morris water maze

The Morris water maze test is a widely accepted method for examining cognitive function. Briefly, a circular plastic pool with a height of 35 cm and a diameter of 100 cm was filled with water maintained at 22–25 °C. An escape platform, measuring 14.5 cm in height and 4.5 cm in diameter, was submerged approximately 1.5 cm below the water surface. After 7 days of as-prepared compounds treatment, a pre-acquisition session was carried out on 8 d for once. The test was conducted 4 times daily for 5 days during the acquisition phase (9–13 d) with randomized starting points. Each trial had a duration of 60 s or concluded when the mice reached the submerged platform. Swimming behavior of each mouse was continuously monitored and recorded by a camera positioned above the center of the pool. Parameters including escape latency, escape distance, and swimming speed were evaluated using the SMART-LD program. Probe trial sessions were held on the last day of the experiment.

### Pharmacokinetics in the rat

An equal number of female and male rats weighing 180–220 g were used in the following studies. Food and water were fed ad libitum throughout the study. After an oral administration of rhein, geraniol, or PPRG at the dose of 35 or 70 mg/kg, blood samples were collected from the tail vein at the following time points 0.25, 0.5, 0.75, 1, 2, 3, 4, 6, 8, 12 and 14 h. The blood was centrifuged at 3000 rpm for 5 min at 4 °C, and then plasma was decanted into separate tubes and stored frozen at -20 °C before drug analysis. A total of 10 µL of 2 mol/L hydrochloric acid was added to a 50 µL plasma sample and thoroughly mixed. Analytes were extracted from the sample using 1 mL of acetic ether. The plasma samples were vortexed for 3 min and then centrifuged at 3500 rpm for 10 min. The organic layers were separated, and the solvent was evaporated until dry at room temperature. The residue was then reconstituted in 100 µL of the mobile phase, and a 10 µL volume was injected into an LC–MS/MS system for analysis.

### Statistical analysis

All of experiments were repeated at least three times. All the results were presented as mean values with standard deviation (SD). Statistical analysis and graphing were used Tukey's Method with one-way ANOVA analysis (Prism 8.0, Graph pad Software Inc., San Diego, CA, USA). Statistical significance is denoted by *P < 0.05, **P < 0.01, and ***P < 0.001.

### Supplementary Information

**Additional file 1: Table S1.** PCR primers for inflammatory factor genes and bacterial identification. **Table S2.** Gel permeation chromatography results for synthetic compounds used in this study.** Figure S1.** Synergism of rhein and geraniol in vitro. (A) Checkerboard assay of rhein and geraniol. (B) Time-killing curves of rhein and geraniol. **Figure S2.** ^1^H NMR and FTIR characteristics of PPG and PPR. Structural formulas of (A) PPR and (B) PPG. ^1^H NMR spectra of (C) PPR and (D) PPG. FTIR spectra of (E) PPR and (F) PPG. **Figure S3.** XPS spectra of the PPR and PPRG. **Figure S4.** The XRD spectra of poly(α-lipoic acid), PPR, PRMG, and rhein. **Figure S5.** Rhein and geraniol release amount from PPRG nanoprodrug in dissolve medium. (A) Rhein release amount from PPR and PPRG nanoprodrugs at pH 7.4 containing 1 mM DTT, (B) Geraniol release amount from PPG and PPRG nanoprodrugs at pH 7.4 containing 1 mM DTT. (C) 14-day room temperature storage stability test of PPRG. **Figure S6.** Cell viability of (A) HGF-1 and (B) Hep G2. (C) Live/dead cell staining of 293 T cells. Test samples were added to cells at 40 µM each. CON, control; SAL, Salmonella; RH, rhein; PPR, PEGylated poly (α-lipoic acid)-grafted rhein; GE, geraniol; PPG, PEGylated poly (α-lipoic acid)-grafted geraniol; PPRG, Poly(α-lipoic acid)-polyethylene glycol grafted rhein and geraniol. **Figure S7.** Apparent drug permeability in Caco-2 cells. (A) Transepithelial electrical resistance (TEER) of Caco-2 cells. (B) The permeability of the rhein, PPR and PPRG compounds. (C) The permeability of the geraniol, PPG and PPRG compounds. **Figure S8.** Intestinal morphology of mice in the indicated test groups. H&E staining of duodenum, ileum, and conlon. **Figure S9.** The performance of the mice in the Morris water maze test. (A) The timeline of drug treatment, spatial acquisition and probe trial sessions of the Morris water maze. (B) The body weight changes during the whole experiment. (C) Representative swimming paths of the mice receiving PBS, rhein, geraniol or PPRG treatment during the probe trial. (D-E) The path length and escape latency in the four groups over six consecutive training days. (F) Platform crossing (G) Passing Times (H) Escape latency on the 7th day of the spatial acquisition session. **Figure S10.** Average plasma concentration-time curve of (A) rhein, (B) PPRG-rhein, (C) geraniol, and (D) PPRG-geraniol after intragastric administration in rats (n=4). **Table S3.** Pharmacokinetic parameters of drugs in rats after oral administration. **Table S4.** Abbreviations.

## Data Availability

The data that support the findings of this study are available from the corresponding author upon reasonable request.
