# Supplementary material for: Self-assembly of H2S-responsive nanoprodrugs based on natural rhein and geraniol for targeted therapy against Salmonella Typhimurium
Source: J Nanobiotechnology. 2023 Dec 16;21:483. doi: 10.1186/s12951-023-02256-9 (PMC10725032; doi:10.1186/s12951-023-02256-9)
Supplement: Supplementary file 1 — Additional file 1: Table S1. PCR primers for inflammatory factor genes and bacterial identification. Table S2. Gel permeation chromatography results for synthetic compounds used in this study. Figure S1. Synergism of rhein and geraniol in vitro. (A) Checkerboard assay of rhein and geraniol. (B) Time-killing curves of rhein and geraniol. Figure S2. 1H NMR and FTIR characteristics of PPG and PPR. Structural formulas of (A) PPR and (B) PPG. 1H NMR spectra of (C) PPR and (D) PPG. FTIR spectra of (E) PPR and (F) PPG. Figure S3. XPS spectra of the PPR and PPRG. Figure S4. The XRD spectra of poly(α-lipoic acid), PPR, PRMG, and rhein. Figure S5. Rhein and geraniol release amount from PPRG nanoprodrug in dissolve medium. (A) Rhein release amount from PPR and PPRG nanoprodrugs at pH 7.4 containing 1 mM DTT, (B) Geraniol release amount from PPG and PPRG nanoprodrugs at pH 7.4 containing 1 mM DTT. (C) 14-day room temperature storage stability test of PPRG. Figure S6. Cell viability of (A) HGF-1 and (B) Hep G2. (C) Live/dead cell staining of 293 T cells. Test samples were added to cells at 40 µM each. CON, control; SAL, Salmonella; RH, rhein; PPR, PEGylated poly (α-lipoic acid)-grafted rhein; GE, geraniol; PPG, PEGylated poly (α-lipoic acid)-grafted geraniol; PPRG, Poly(α-lipoic acid)-polyethylene glycol grafted rhein and geraniol. Figure S7. Apparent drug permeability in Caco-2 cells. (A) Transepithelial electrical resistance (TEER) of Caco-2 cells. (B) The permeability of the rhein, PPR and PPRG compounds. (C) The permeability of the geraniol, PPG and PPRG compounds. Figure S8. Intestinal morphology of mice in the indicated test groups. H&E staining of duodenum, ileum, and conlon. Figure S9. The performance of the mice in the Morris water maze test. (A) The timeline of drug treatment, spatial acquisition and probe trial sessions of the Morris water maze. (B) The body weight changes during the whole experiment. (C) Representative swimming paths of the mice receiving PBS, rhein, [file 12951_2023_2256_MOESM1_ESM.docx]

**Additional file**

**Self-assembly of H2S-Responsive Nanoprodrugs based on natural Rhein and Geraniol for Targeted Therapy against *Salmonella Typhimurium***

Lu Han^1, 2, 4^ †, Tao Zang^1, 4^ †, Lu-Lu Tan^1, 4^, Dun-Sheng Liang^1, 4^, Teng-Fei Long^1, 4^, Xu-Wei Liu^1, 4^, Xiao-Fan Shen^1, 4^, Hao Ren^1, 4, 5^, Zhi-Peng Li^1, 4^, Zhao-Xiang Lu^1, 4^, Sheng-Qiu Tang^2^, Xiao-Ping Liao^1, 4, 5^, Ya-Hong Liu^1, 4, 5^, Chao-Qun Zhang^3*^, Jian Sun^1, 4, 5*^

^1^State Key Laboratory for Animal Disease Control and Prevention, South China Agricultural University, Guangzhou, China;

^2^Guangdong Provincial Key Laboratory of Utilization and Conservation of Food and Medicinal Resources in Northern Region, Henry Fok School of Biology and Agriculture, Shaoguan University, Shaoguan 512005, PR China;

^3^Key Laboratory for Biobased Materials and Energy of Ministry of Education, College of Materials and Energy, South China Agricultural University, Guangzhou 510642, PR China;

^4^Guangdong Provincial Key Laboratory of Veterinary Pharmaceutics, Development and Safety Evaluation, South China Agricultural University, Guangzhou 510642, PR China;

^5^Jiangsu Co-Innovation Center for the Prevention and Control of Important Animal Infectious Diseases and Zoonoses, Yangzhou University, Yangzhou 225009, PR China;

†These authors contributed equally to this work.

*To whom correspondence should be addressed:

[zhangcq@scau.edu.cn](mailto:zhangcq@scau.edu.cn) (Chaoqun Zhang)

[jiansun@scau.edu.cn](mailto:jiansun@scau.edu.cn) (Jian Sun)

**1. Chemical** **reagents**

Alpha-lipoic acid (ALA), 1-(3-dimethylaminopropyl)-3-ethylcarbodiimide hydrochloride (EDC·HCl), dithiothreitol (DTT) were purchased from Yuanye Biological Technology (Shanghai, China). Polyethylene glycol monomethyl ether 2000, rhein, geraniol, chloroform-d, (methyl sulfoxide)-d_6_, hematoxylin and eosin were ordered from Macklin Biochemical Technology (Shanghai, China). N, N-Dimethylformamide (DMF), sodium sulfide nonahydrate (Na_2_S**·**9H_2_O), 4-dimethylaminopyridine (DMAP), tetrahydrofuran (THF) and dimethyl sulfoxide (DMSO) were obtained from Aladdin Biochemical (Shanghai, China). Cell Counting Kit-8 (CCK-8) and Calcein/PI Cell Viability/Cytotoxicity Assay Kit were acquired from Beyotime Biotechnology (Beijing, China). Interleukin-6 (IL-6), Interleukin-10 (IL-10), tumor necrosis factor-α (TNF-α) ELISA kits for mouse were purchased from Biological Technology co., Ltd (Wuhan, China). Dulbecco's modified Eagle's medium (DMEM, Gibco), phosphate buffer (PBS) and fetal bovine serum (FBS, Gibco) was ordered from Thermo Fisher Scientific (Shanghai, China). Luria-Bertani (LB) broth and anaerobic broth were obtained from Guangzhou Huankai Microbial Technology (Guangzhou, China). RNA extraction kits were purchased from Accurate Biology (Changsha, China), and reverse transcription and real-time SYBR Green kits were purchased from Vazyme Biotech (Nanjing, China). The human intestinal epithelial cell lines (Caco-2), human embryonic kidney cells (293T), murine macrophage RAW 264.7 (RAW 264.7), human hepatocellular carcinoma cells (Hep G2), and human gingival fibroblasts cells (HGF-1) were obtained from the cell bank of the type culture collection of the Chinese Academy of Sciences (Shanghai, China). *S. Typhimurium* ATCC 14028 was purchased from China general microbiological culture collection center (GMCC, China).

**2. Characterization**

The H proton magnetic resonance (^1^H NMR) spectra were recorded on a Bruker AV 600M spectrometer (Karlsruhe, Germany). Fourier transform infrared spectroscopy (FTIR) were performed on a Thermo Fisher Nicolet IS10 FTIR spectrophotometer (Waltham, MA, USA). Gel permeation chromatography (GPC) tests were performed in DMF solutions at 35°C with an elution rate of 1.0 mL**·**min^-1^ using a Waters 1525 system equipped with a Waters 2414 detector (Burlington, MA, USA) that was calibrated using polystyrene standards ranging from 200 to 500,000 g/mol. The particle sizes were characterized using a Zeta-sizer Nano ZSE instrument (Malvern, Worcestershire UK). The bonding characterization and morphology of the samples were carried out via X-ray photoelectron spectroscopy (XPS), X-ray diffraction (XRD) and transmission electron microscopy (TEM) using Thermo Scientific Nexsa G2 (Waltham, MA, USA), Rigaku MiniFlex600 (Tokyo, Japan) and Thermo Fisher FEI/Talos L120C (Waltham, MA, USA), respectively. High performance liquid chromatograph (HPLC) measurements were performed using a Shimadzu LC-AT20 (Kyoto, Japan). Cell images were captured using DMI8 Leica fluorescence inverted microscope (Wetzlar, Germany). Histological sections were stained with hematoxylin and eosin (H&E) and observed using a Nikon TE2000U optical microscope (Tokyo, Japan). Fluorescent intensity and optical density (OD) measurements were performed with a PerkinElmer PHERAstar FS microplate reader (Waltham, MA, USA). Inflammatory factors were determined using fluorescence quantitative polymerase chain reaction (qPCR) on a BioRad CFX instrument (Hercules, CA, USA) and reverse transcription (RT) reactions were performed using a SensoQuest system (Gottingen, Germany).

**3. The ^1^H NMR of PPR and PPG analysis**

The rhein proton peaks h_2_, i_2_, j_2_, k_2_, l_2_ (δ7.36, δ7.68, δ7.70, δ7.79, δ7.84 and δ8.10 ppm, respectively) appeared in the ^1^H NMR spectra of the PR, indicating that PR was synthesized successfully (Fig. S2d). The new proton signals (h, i, j, k and l) appeared at δ7.39, δ7.70, δ7.81, δ7.84, δ8.10 ppm, respectively, indicating the presence of a benzene ring in the ^1^H NMR spectra of PPR. Meanwhile, the peaks corresponding to the phenolic hydroxyl group at δ11.87 ppm disappeared, suggesting that the hydroxyl group had undergone a chemical reaction or had been replaced by another functional group (Fig. 1B). The structures of PR and PPR were confirmed by FTIR and GPC analyses. In PPR, the intensity of the hydroxyl (-OH) peak at 3133.26 cm^-1^ was much higher than that observed in PALA and PR, indicating the presence of more hydroxyl groups in the polymer (Fig. S2E). The molecular weights of PR and PPR were found to be 54130 and 61895 and 64657 g·mol^-1^, respectively, indicating successful grafting of 10.4% rhein and 0.38% PEG onto the PPR structure (Fig. 1d, Table S2). These results showed that PPR was successfully synthesized.

The ^1^H NMR spectra of PPG show new proton signals (δ3.91, δ5.26, δ1.72, δ2.03, δ2.06, δ5.09, δ1.95 ppm) for the m_1_, n_1_, o_1_, p_1_, q_1_, r_1_, s_1_, and t_1_ positions in the PEG-PALA skeleton. Additionally, new proton peaks (h_1_, i_1_, j_1_, k_1_, l_1_, m_1_, n_1_, o_1_) appeared in the PPG structure at δ4.75, δ5.23, δ1.56, δ1.94, δ1.94, δ4.98, δ1.56, and δ1.56 ppm. The proton at δ4.45 ppm of the geraniol hydroxyl disappeared, suggesting that the hydroxyl group had undergone a chemical reaction or had been replaced by another functional group (Fig.1B). In addition, GPC analysis showed that the average weight M_w_ of PPG was 61457 g·mol^-1^, greater than PEG-PALA at 55227 g·mol^-1^, indicating that 15.4% geraniol was grafted onto the PALA skeleton structure. These results indicated that PPG was successfully synthesized.

**Table S1 PCR primers for inflammatory factor genes and bacterial identification**

| Name | Sequence (5’ to 3’) |
| --- | --- |
| TNF-α-F | CTCTTCAAGGGACAAGGCTG |
| TNF-α-R | CGGACTCCGCAAAGTCTAAG |
| IL-6-F | CCGGAGAGGAGACTTCACAG |
| IL-6-R | TCCACGATTTCCCAGAGAAC |
| IL-1β-F | GACCTTCCAGGATGAGGACA |
| IL-1β-R | AGGCCACAGGTATTTTGTCG |
| GAPDH-F | ACCCAGAAGACTGTGGATGG |
| GAPDH-R | CACATTGGGGGTAGGAACAC |
| IL-10-F | GCTCCTAGAGCTGCGGACT |
| IL-10-R | TGTTGTCCAGCTGGTCCTTT |

**Table S2** **Gel permeation chromatography results for synthetic compounds used in this study**

| Name | M_W_ | PDI |
| --- | --- | --- |
| PALA | 54130 | 2.39 |
| mPEG | 2762 | 1.4 |
| PALA-mPEG | 55227 | 1.87 |
| PALA-RH | 61895 | 1.80 |
| PALA-mPEG-GE | 61457 | 2.06 |
| PALA-RH-mPEG | 64657 | 1.85 |
| PALA-RH-mPEG-GE | 88674 | 1.80 |

**Figure S1**

**
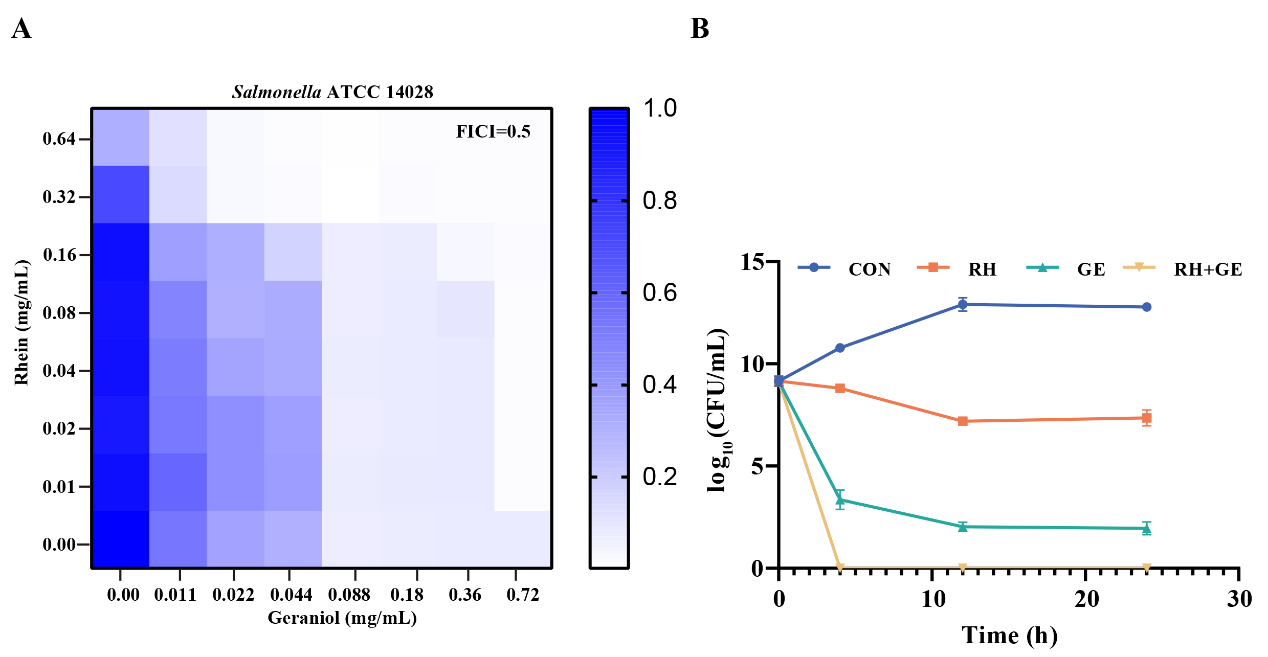
**

**Figure S1** Synergism of rhein and geraniol *in vitro*. (A) Checkerboard assay of rhein and geraniol. (B) Time-killing curves of rhein and geraniol.

**Figure S2**

**
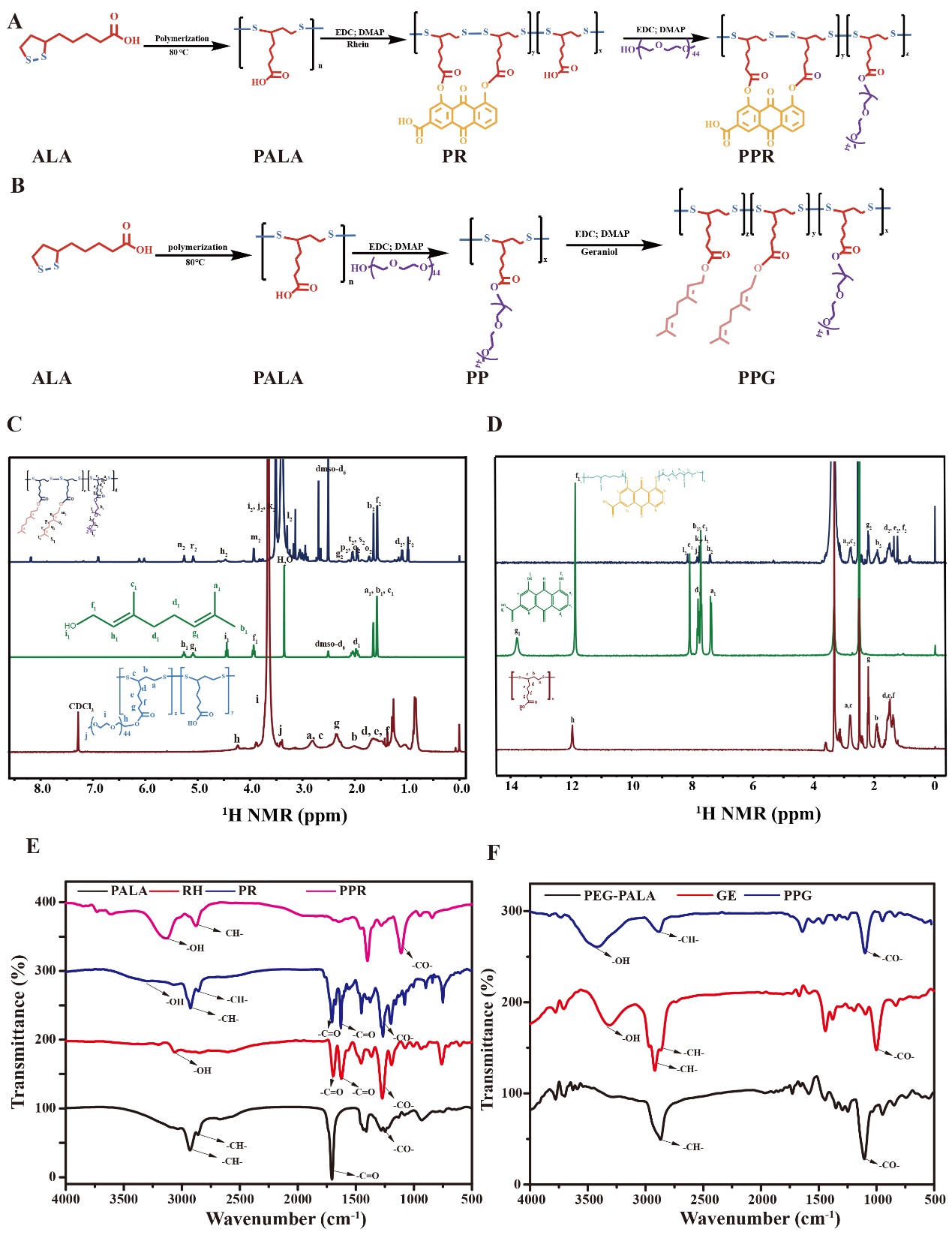
**

**Figure S2** ^1^H NMR and FTIR characteristics of PPG and PPR. Structural formulas of (A) PPR and (B) PPG. ^1^H NMR spectra of (C) PPR and (D) PPG. FTIR spectra of (E) PPR and (F) PPG.

**Figure S3**


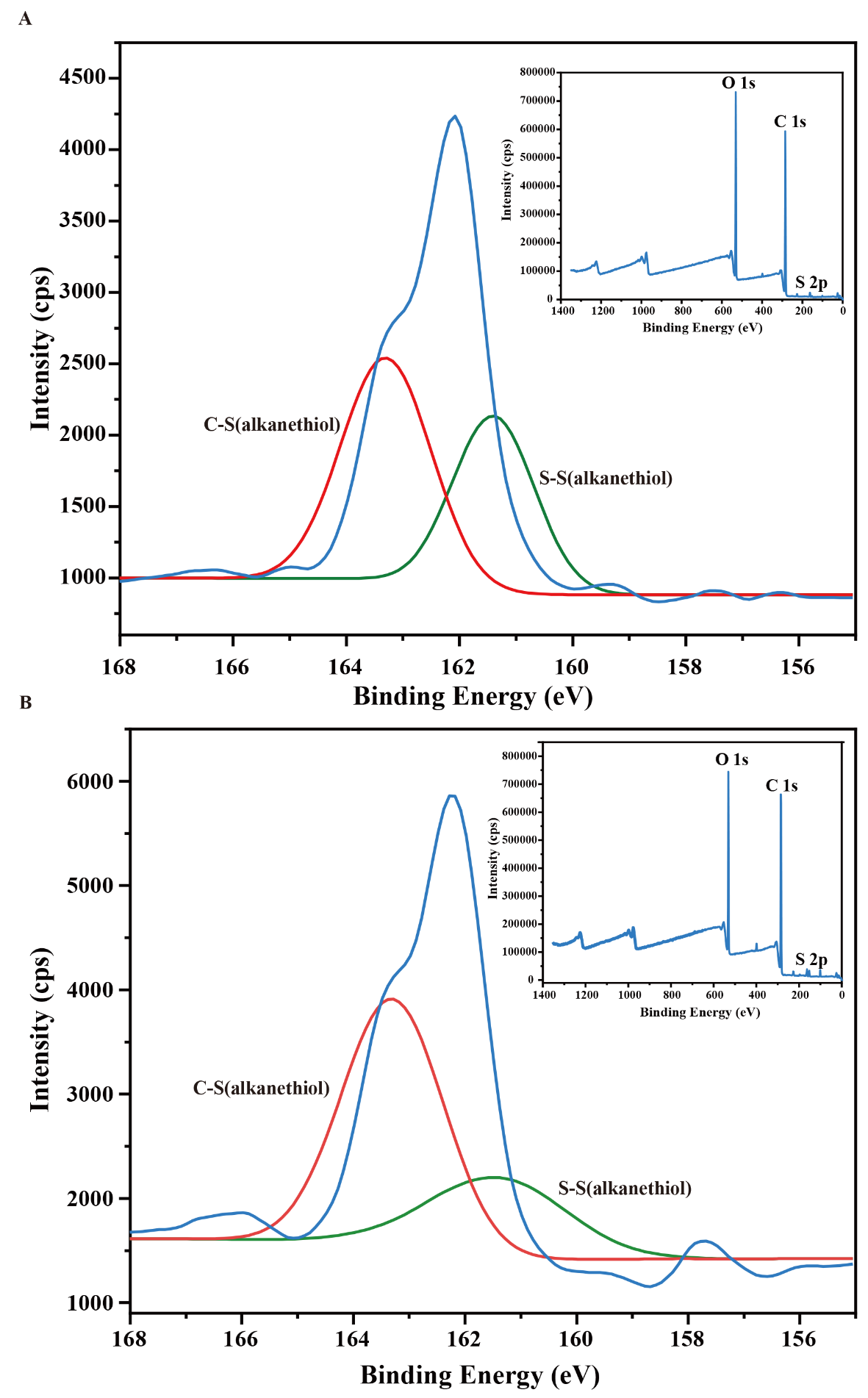


**Figure S3** XPS spectra of the PPR and PPRG.

**Figure S4**

**
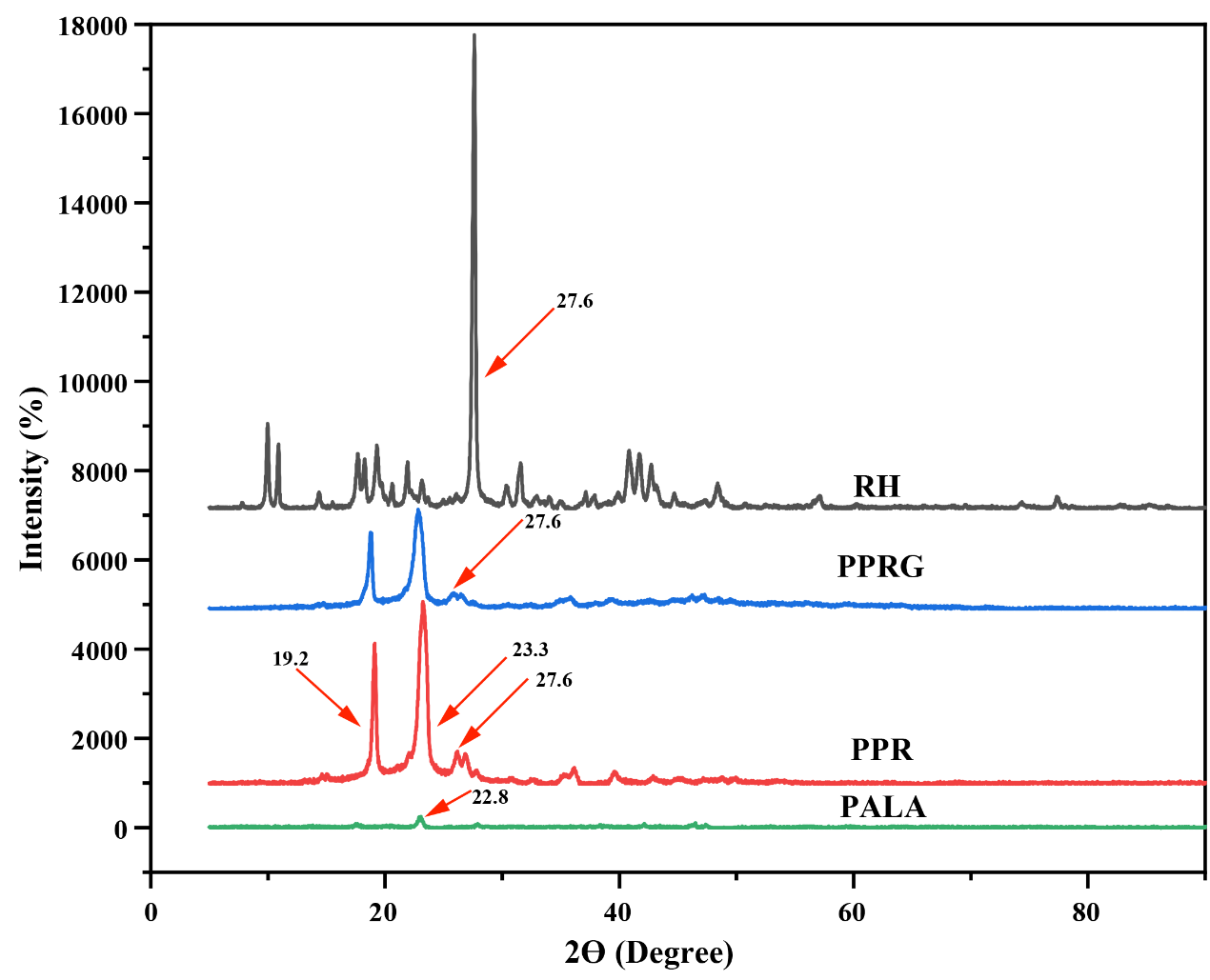
**

**Figure S4.** The XRD spectra of poly(α-lipoic acid), PPR, PRMG, and rhein.

**Figure S5**

**
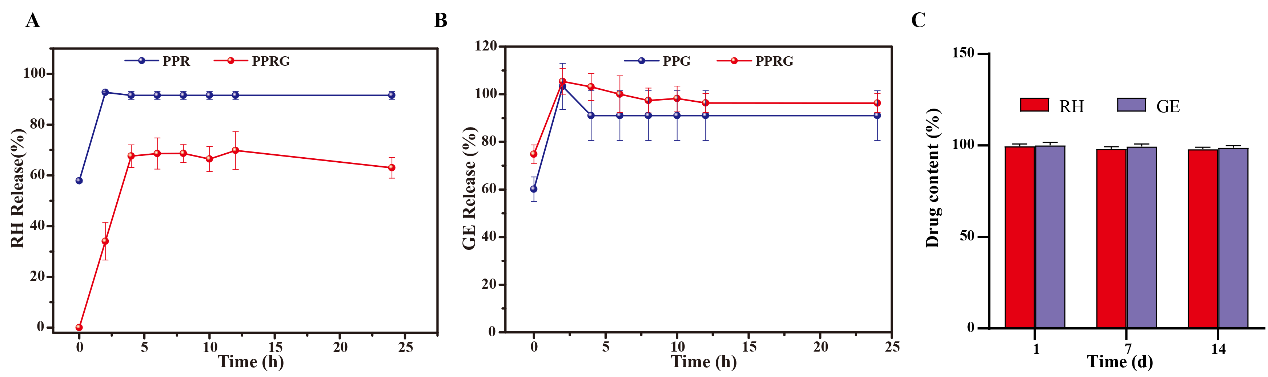
**

**Figure S5** Rhein and geraniol release amount from PPRG nanoprodrug in dissolve medium. (A) Rhein release amount from PPR and PPRG nanoprodrugs at pH 7.4 containing 1 mM DTT, (B) Geraniol release amount from PPG and PPRG nanoprodrugs at pH 7.4 containing 1 mM DTT. (C) 14-day room temperature storage stability test of PPRG.

**Figure S6**

**
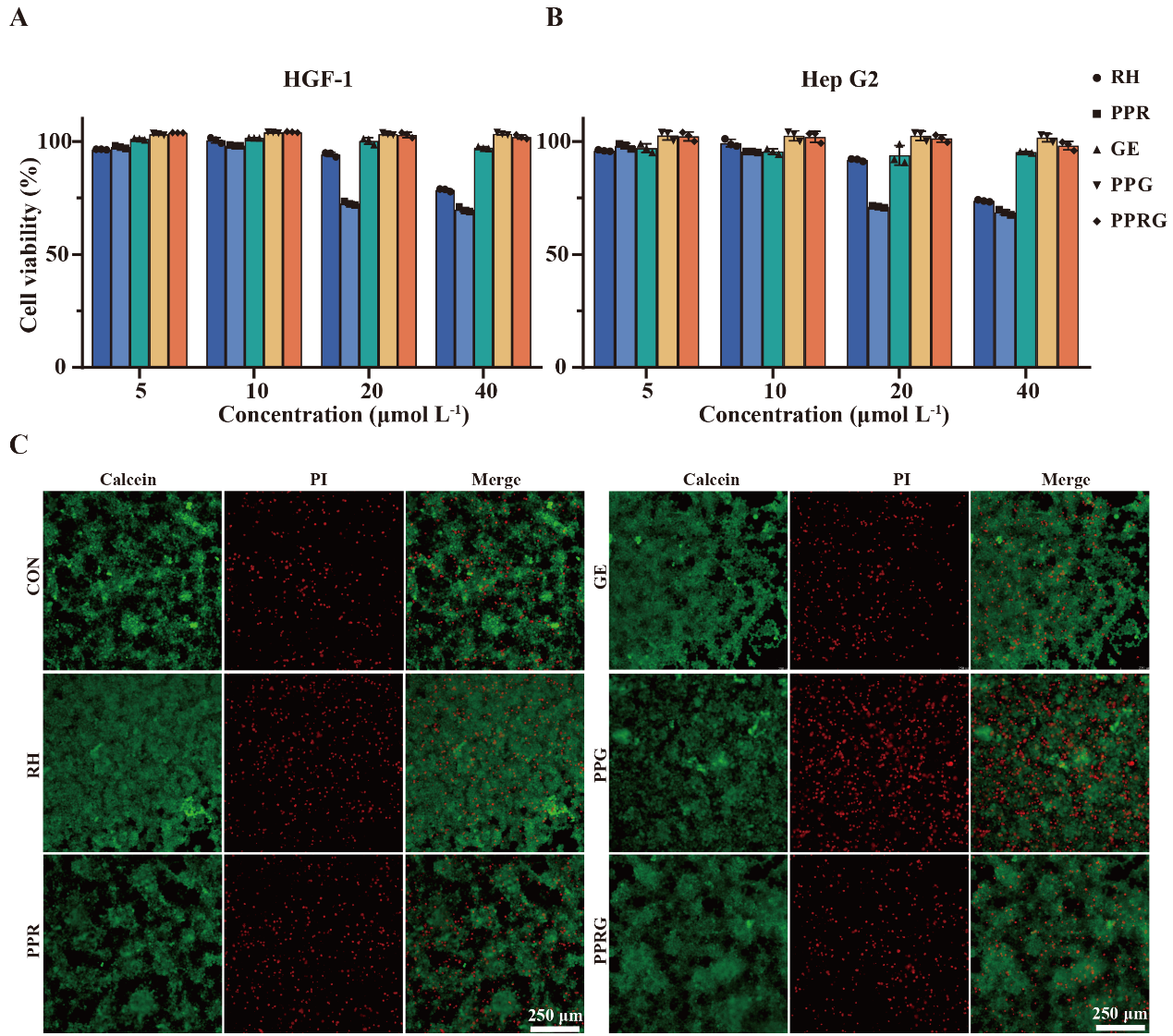
**

**Figure S6.** Cell viability of (A) HGF-1 and (B) Hep G2. (C) Live/dead cell staining of 293T cells. Test samples were added to cells at 40 µM each. CON, control; SAL, *Salmonella;* RH, rhein; PPR, PEGylated poly (α-lipoic acid)-grafted rhein; GE, geraniol; PPG, PEGylated poly (α-lipoic acid)-grafted geraniol; PPRG, Poly(α-lipoic acid)-polyethylene glycol grafted rhein and geraniol.

**Figure S7**


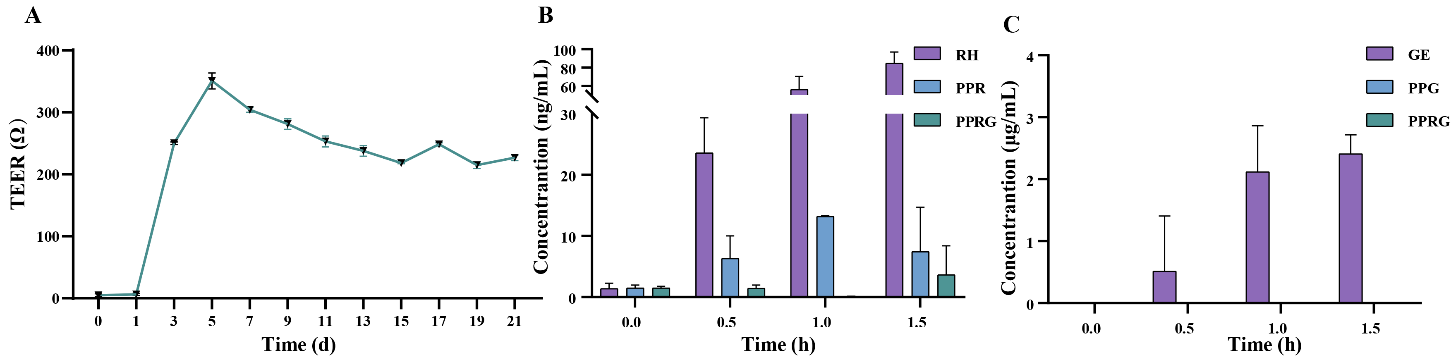


**Figure S7** Apparent drug permeability in Caco-2 cells. (A) Transepithelial electrical resistance (TEER) of Caco-2 cells. (B) The permeability of the rhein PR and PRG compounds.

**Figure S8**


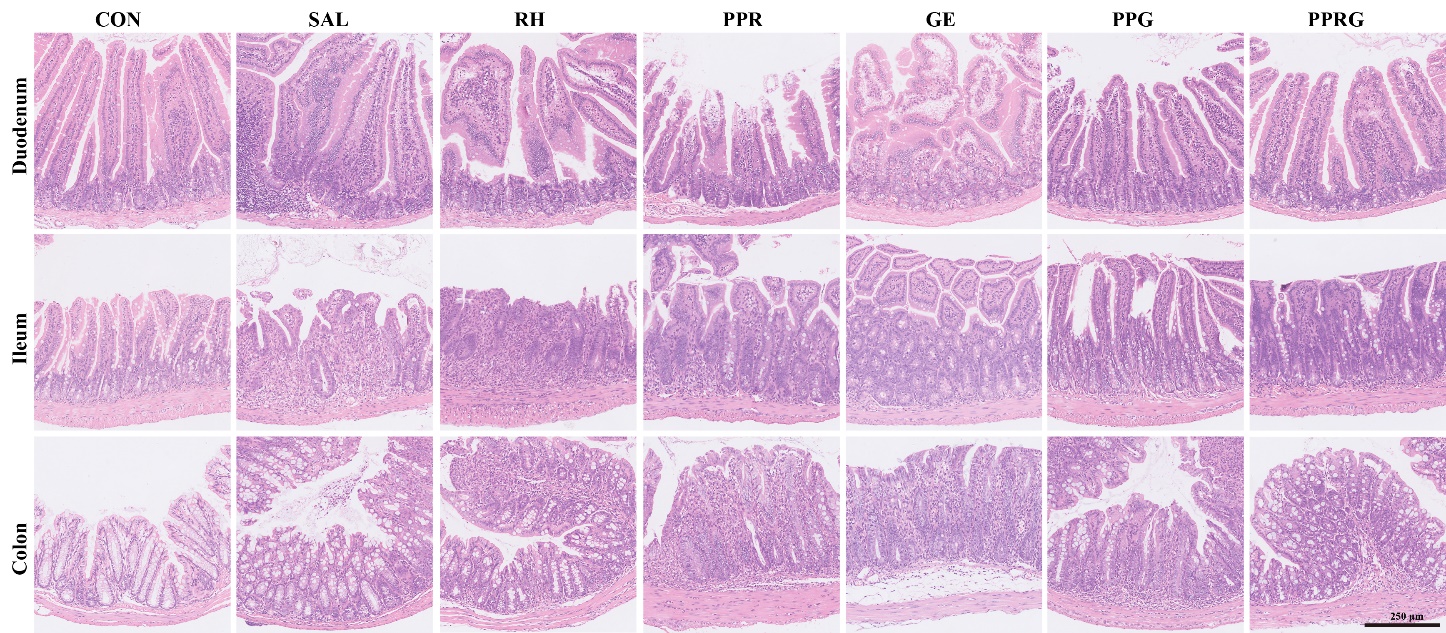


**Figure S8** Intestinal morphology of mice in the indicated test groups. H&E staining of duodenum, ileum, and conlon.

**Figure S9**


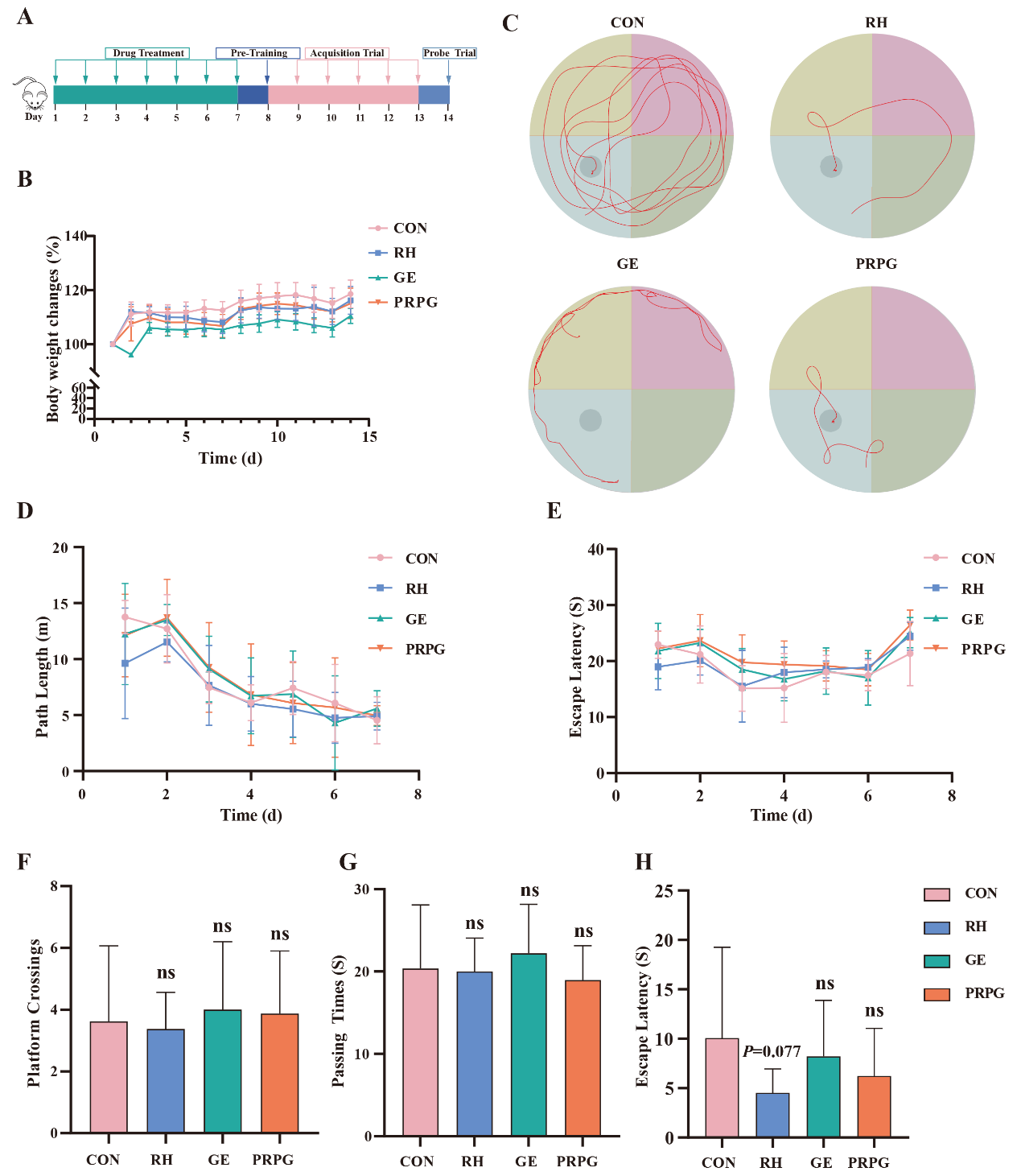


**Figure S9.** The performance of the mice in the Morris water maze test. (A) The timeline of drug treatment, spatial acquisition and probe trial sessions of the Morris water maze. (B) The body weight changes during the whole experiment. (C) Representative swimming paths of the mice receiving PBS, rhein, geraniol or PPRG treatment during the probe trial. (D-E) The path length and escape latency in the four groups over six consecutive training days. (F) Platform crossing (G) Passing Times (H) Escape latency on the 7th day of the spatial acquisition session.

**Figure S10.**

**
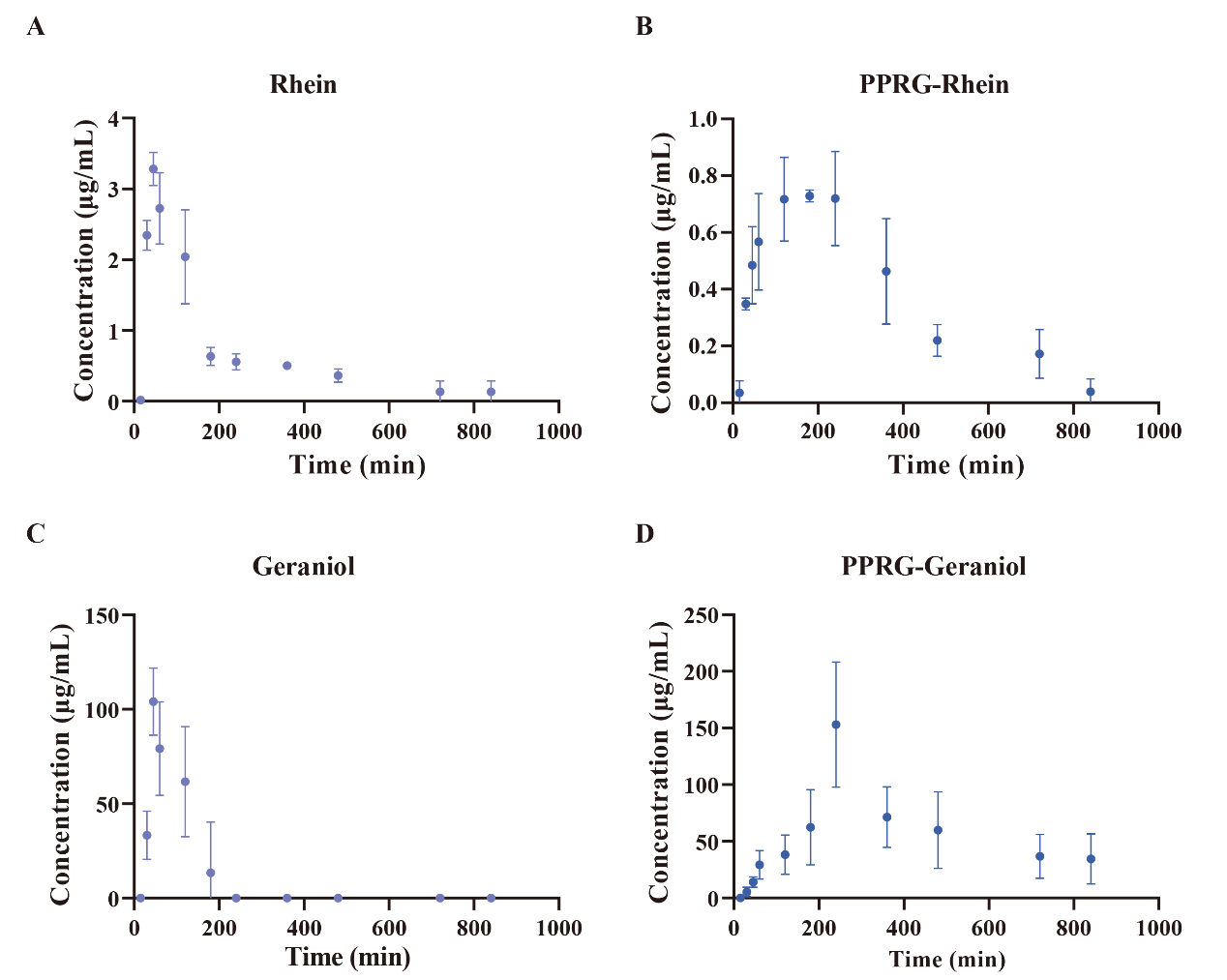
**

**Figure S10.** Average plasma concentration-time curve of (A) rhein, (B) PPRG-rhein, (C) geraniol, and (D) PPRG after intragastric administration in rats (n=4).

**Table S3 Pharmacokinetic parameters of drugs in rats after oral administration.**

| Parameters | GE | RH | PPRG | |
| --- | --- | --- | --- | --- |
|  |  |  | GE | RH |
| t_max_ (h) | 0.75 | 0.75 | 4.5 ± 0.75 | 3 ± 1 |
| C_max_ (µg/mL) | 104.12 ± 11.35 | 3.28 ± 0.17 | 155.20 ± 43.9 | 0.85 ± 0.03 |
| MRT (h) | 1.25 ± 0.17 | 3.27 ± 0.56 | 6.29 ± 0.85 | 4.86 ± 0.60 |
| t_1/2_ (h) | 2.04 ± 0.96 | 1.61 ± 0.35 | 7.88 ± 1.78 | 3.25 ± 0.20 |
| AUC (µg/mL/L) | 329.93 ± 185.78 | 25.61 ± 18.88 | 1216.23 ± 397.82 | 5.73 ± 0.53 |
| CL (L/kg/h) | 0.31 ± 0.17 | 4.09 ± 0.71 | 0.064 ± 0.021 | 6.20 ± 0.51 |
| V (L/kg) | 0.67 ± 0.075 | 9.14 ± 0.42 | 0.7 ± 0.23 | 29.05 ± 2.96 |

**Table S4. Abbreviations**

| **Abbreviations** | **Full Name** |
| --- | --- |
| ^1^H NMR | ^1^H nuclear magnetic resonance |
| 293T | Human Renal Epithelial Cells |
| ALA | α-lipoic acid |
| ApisOBP9 | Aphid odorant-binding protein 9 |
| Caco-2 | Human colorectal adenocarcinoma cells |
| DMAP | 4-Dimethylaminopyridine |
| DMEM | Dulbecco's Modified Eagle Medium |
| DMF | N, N-dimethylformamide |
| DMSO | Dimethyl sulfoxide |
| DTT | dithiothreitol |
| EDC·HCl | 1-(3-Dimethylaminopropyl)-3-ethylcarbodiimide hydrochloride |
| FBS | Fetal bovine serum |
| FTIR | Fourier transform infrared spectrum |
| GPC | Gel permeation chromatography |
| H&E | hematoxylin-eosin |
| H_2_S | Hydrogen sulfide |
| HPLC | High Performance Liquid Chromatography |
| IL-10 | Interleukin-10 |
| IL-6 | Interleukin-6 |
| IL-1β | Interleukin-1β |
| LPS | Lipopolysaccharide |
| MIC | Minimal Inhibitory Concentration |
| mRNA | Messenger RNA |
| Na_2_S | Sodium sulfide |
| NO | Nitric oxide |
| OD | Optical Density |
| PALA | Poly (α-lipoic acid) |
| PBS | Phosphatic buffer solution |
| PEG | Monomethoxy poly ethylene glycol |
| PPG | PEGylated PALA grafted geraniol |
| PR | Rhein grafting Poly (α-lipoic acid) |
| PPR | PEGylated PALA grafted rhein |
| **Abbreviations** | **Full Name** |
| PPRG | Poly(α-lipoic acid)-polyethylene glycol grafted rhein and geraniol |
| RAW 264.7 | Mouse mononuclear macrophage |
| RT-PCR | Real-Time PCR |
| SGF | Simulated gastric fluid |
| SIF | Simulated intestinal fluid |
| TEER | Trans-Epithelial Electrical Resistance |
| TEM | Transmission electron microscope |
| THF | Tetrahydrofuran |
| TNF-α | Tumor necrosis factor α |
